# Investigating the Impact of Hydrophobic Polymer Segments on the Self-Assembly Behavior of Supramolecular Cyclic Peptide Systems via Asymmetric-Flow Field Flow Fractionation

**DOI:** 10.1021/acs.macromol.3c00442

**Published:** 2023-08-26

**Authors:** Maria Kariuki, Julia Y. Rho, Stephen C. L. Hall, Sébastien Perrier

**Affiliations:** † Department of Chemistry, University of Warwick, Coventry CV4 7AL, U.K.; ‡ ISIS Neutron and Muon Source, Rutherford Appleton Laboratory, Didcot OX11 0QX, U.K.; § Warwick Medical School, University of Warwick, Coventry CV4 7AL, U.K.; ∥ Faculty of Pharmacy and Pharmaceutical Sciences, Monash University, Parkville, VIC 3052, Australia

## Abstract

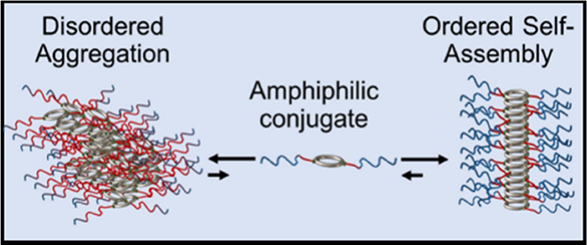

The present study examines the behavior of cyclic peptide polymer conjugates that have been designed to combine their self-assembling ability via H-bonding with the properties of amphiphilic diblock copolymers. Using a combination of asymmetric flow-field flow fractionation (AF_4_) and small-angle neutron scattering (SANS), we have uncovered unique insight based on the population of structures established at a 24 h equilibrium profile. Our results determine that by introducing a small quantity of hydrophobicity into the conjugated polymer corona, the resulting nanotube structures exhibit low unimer dissociation which signifies enhanced stability. Furthermore, as the hydrophobicity of the polymer corona is increased, the elongation of the nanotubes is observed due to an increase in the association of unimers. This encompasses not only the H-bonding of unimers into nanotubes but also the self-assembly of single nanotubes into segmented-nanotube structures with high aspect ratios. However, this influence relies on a subtle balance between the hydrophobicity and hydrophilicity of the polymer corona. This balance is proposed to determine the solvent entropic penalty of hydrating the system, whereby the cost scales with the hydrophobic quantity. Consequently, it has been suggested that at a critical hydrophobic quantity, the solvation penalty becomes high enough such that the self-assembly of the system deviates from ordered hydrogen bonding. The association behavior is instead dominated by the hydrophobic effect which results in the undesirable formation of disordered aggregates.

## 1 Introduction

A common practice in supramolecular chemistry is the incorporation of multiple noncovalent interactions which synergistically provide control over the size, stability, and functionality of supramolecular assemblies.^1^ Within the context of self-assembly in aqueous environments, distinctive structures have been developed by the synergistic cooperation between directional hydrogen (H−) bonds and interactions arising from the hydrophobic effect.^1−5^ For example, peptide amphiphiles combine the directionality of intermolecular hydrogen bonding among β-sheet peptide segments and the hydrophobic collapse of alkyl tails to govern the morphology of resulting assemblies.^6−8^ Hydrophobic stabilization of H-bonded supramolecular motifs has also been essential in progressing synthetic strategies such as living seeded polymerization which enables the control of structural parameters.^1,2,9−13^ Yamaguchi and coauthors, for instance, demonstrated the significance of the hydrophobic effect in creating metastable folded structures required to regulate the spontaneous assembly of their cystine-based diamide monomer (PyC).^10^

Owing to the many benefits achievable through this 2-fold self-assembly approach, our group has recently exploited the complementarity of H-bonding and amphiphilic interactions to engineer complex supramolecular assemblies based on a α-alt(d, l)-cyclic peptide scaffold.^14−16^ These cyclic peptides (CPs) exhibit a low energy flat ring conformation, whereby the amine and carbonyl groups of their backbone amide bonds orient perpendicularly to the ring plane. Adjacent cyclic peptide units are thus able to form an extensive network of hydrogen bonds resulting in the formation of nanotubes via β-sheet stacking.^17,18^ The asymmetric conjugation (opposite ends) of hydrophobic and hydrophilic polymer arms was suggested to lead to the formation of secondary assemblies referred to as tubisomes which are a barrel-shaped arrangement of several nanotubes.^15,16^ In this arrangement, H-bonding between the cyclic peptides drives the formation of single nanotubes while the hydrophobic arms trigger a phase separation due to the hydrophobic effect, resulting in a “folded” conformation. In addition to their unique morphology, the tubisomes showed good biocompatibility, high drug loading content, and the potential to disrupt a lysosome membrane or create transmembrane channels that facilitate the escape of molecules.^15,16^

The conjugation of amphiphilic diblock copolymers has also been explored as a means to moderate the highly dynamic behavior of CP-polymer nanotubes (SCPPNs) in aqueous environments, whereby a fast exchange of unimers between nanotubes has been observed.^14,19^ This exchange behavior is propagated by water molecules that compete for H-bond sites with the backbone amide groups of the peptide, consequently disrupting the intermolecular β-sheet hydrogen-bonding network. It was therefore proposed that conjugating diblock amphiphilic polymers to the cyclic peptide, such that the hydrophobic block is peripheral to the core, would stabilize the nanotube structures by excluding water molecules from the intermolecular core interactions. Importantly, the solubility of the SCPPNs would still be maintained due to the presence of an outer hydrophilic shell. This stabilization hypothesis was initially investigated using Förster resonance energy transfer (FRET) to compare the rate of unimer exchange between SCPPNs consisting solely of hydrophilic poly(dimethyl acrylamide, pDMA) arms against those designed with poly(butyl acrylate)-*b-*poly(dimethyl acrylamide) arms.^14^ The results demonstrated that the inclusion of the hydrophobic butyl acrylate block (pBA) afforded a much less dynamic system by substantially attenuating the rate of unimer exchange. This was indicated by differences in the equilibration time scales (an hour vs a week) and degree of mixing (90% vs 41%) of the two conjugate designs. Stochastic optical reconstruction microscopy (STORM) also revealed that incorporating the hydrophobic inner layer promoted further stacking of the stabilized nanotubes into high aspect ratio assemblies. This was postulated to be the direct consequence of lowering the dynamic behavior of the cyclic peptide system.^14^

Herein, we present a comprehensive study in which the mechanistic contribution(s) of the hydrophobic block is further investigated through a structural distribution lens. This involved not only a comparison of CP systems with hydrophilic and amphiphilic arms but also a systematic assessment of the influence of the hydrophobic block’s molecular design. The structural distribution was determined by combining molecular weight distribution data, which distinguishes between unassembled (unimers) and aggregated structures, with complementary structural analyses that describe the morphology of the species within the distribution. We found that this approach ensured that the growth of the systems could be evaluated with less averaging bias.^20−22^ Furthermore, the structural characterization helped identify when the self-assembly was driven by β-sheet stacking based on the presence of nanotubes. With regard to the employed techniques, structural characterization was conducted using small angle neutron scattering (SANS) whereas, for the first time, measurement of the distribution was performed using asymmetric-flow field flow fractionation coupled with multiangle light scattering and refractive index detection (AF_4_-MALS-RI). AF_4_ is a gentle separation technique that resolves sample constituents by driving them into different parabolic laminar flow velocities of a narrow open channel using a fluid stream (crossflow).^23−26^ This is predominantly according to their diffusion coefficients, as determined by their hydrodynamic radius. In summary, smaller molecules equilibrate in regions of fast flow velocity due to their higher coefficients and thus elute earlier in a process referred to as normal elution mode.^23−26^

AF_4_ has been scarcely used to examine supramolecular systems, yet we found that it is a versatile technique for determining the distribution of supramolecular systems. A significant advantage offered by AF_4_ for the characterization of CP systems is the minimized risk of compromising their noncovalent bonds which would cause the systems to dissociate or reorganize, hence falsify their true distribution.^20,21,27^ This is because its open channel architecture appreciably minimizes the presence of destructive flow-induced forces, such as shear and extensional forces, in comparison to the packed columns employed in chromatography.^28−34^ An additional benefit is that the distribution of our systems in-solution could be characterized at a low risk of excluding either small or high *M*_w_ fractions, such as in microscopy and SEC, respectively. In the latter case, this is attributed to the fixed separation ranges as well as the discussed flow forces.^34,35^ With respect to conventional microscopy techniques such as transmission electron microscopy (TEM) and atomic force microscopy (AFM), the resolution scales attainable for these systems in aqueous solutions greatly lags behind whereby the resolution of unimers (Å) is yet to be achieved.^14−16,19,36,37^ Moreover, in relation to electron microscopy, the low electron densities of the peptides result in poor sample-background contrast which significantly limits their resolution. This was the case in this study, whereby the low contrast and relatively small size scales of the formed assemblies resulted in micrographs with poor resolution, making it difficult to draw reliable conclusions. Lastly, the use of AF_4_ required no sample treatments such as filtration, vitrification, or substrate adsorption which could all misrepresent true distribution or behavior of the systems.

## 2 Results and Discussion

### 2.1 Conjugate Design

**Figure 1:**
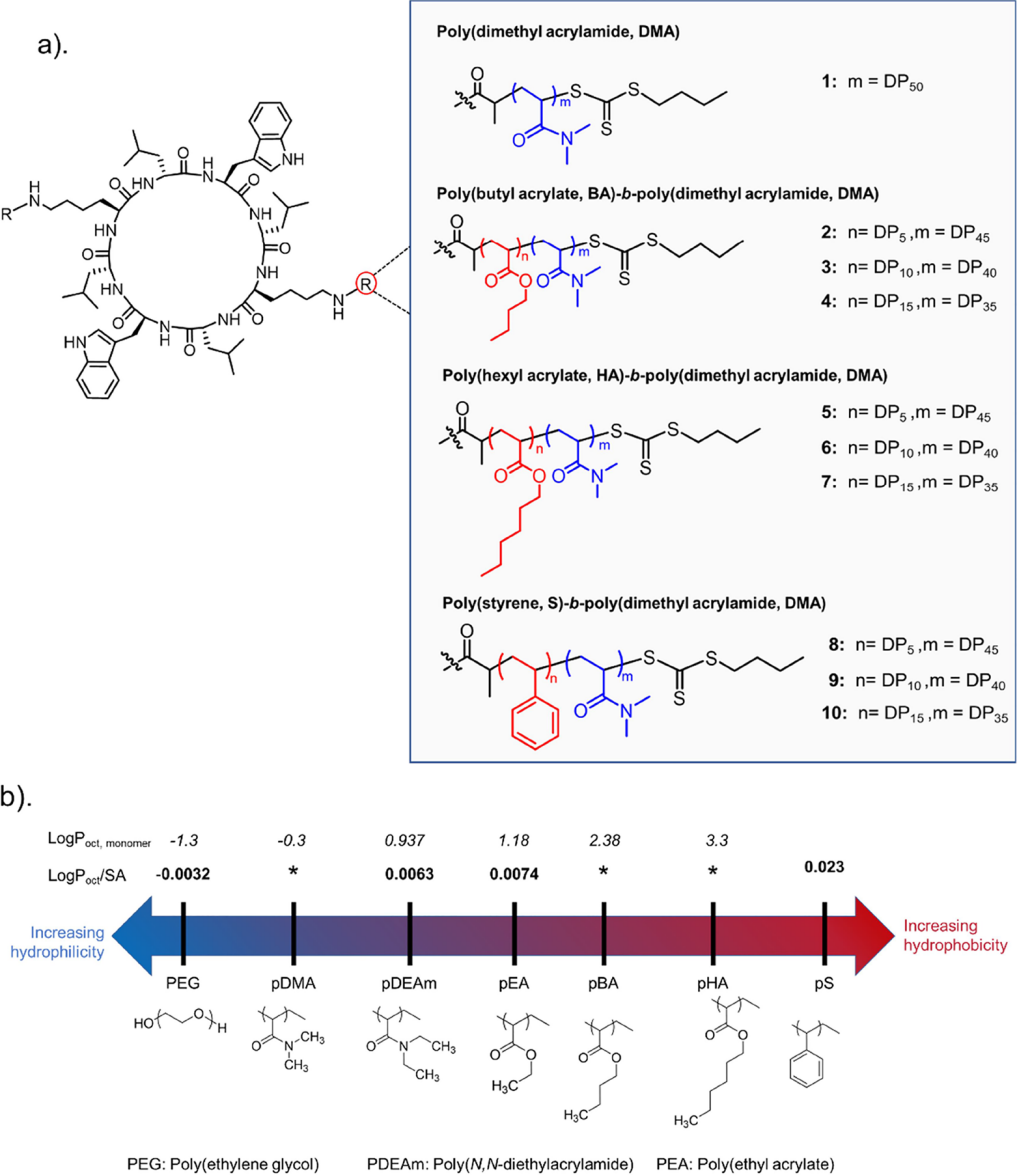
(a) Chemical structures of the cyclic peptide polymer conjugates investigated in this study. The α-alt(d, l)-cyclic peptide core of the conjugates is designed with the following amino acid sequence: (d-Leu-l-Trp-d-Leu-l-Lys-)_2._ (b) Polymer hydrophobicity ranked using MHP (Log*P*_oct_/SA, units: Å^–2^) or Log*P*_oct_ predictive models. MHP values were determined by O’Reilly and co-workers whereas Log*P*_oct_ are referenced from Sigma-Aldrich Safety Data Sheets.^43,44^ Interpretation of the values is based on their sign, where more negative values denote increasing hydrophilicity, whereas more positive values indicate increasing hydrophobicity.

Our previous work indicated that including a hydrophobic block drastically reduced the unimer exchange between SCPPNs and promoted their intermolecular association. However, the relevance of the length and monomer of the hydrophobic block is yet to be investigated.^14^ Therefore, with the aim of establishing a clear correlation between the design of the hydrophobic inner layer and changes to the system behavior, a series of ten bespoke conjugates were synthesized to validate against previous data (Figure 1a). In these designs, the total chain length of the conjugated polymers was kept constant while rationally increasing the hydrophobic content by changing the hydrophobic monomer and/or the hydrophobic to hydrophilic block ratios. This ensured that any observed differences could be solely related to the hydrophobicity, and not due to the polymer length, which has previously been shown to have a steric influence on the CP self-assembly process.^38−42^

Fundamental research by the O’Reilly group regarding hydrophobicity was taken into consideration when comparing the quantitative hydrophobicity of the selected polymer designs.^43,44^ In their work, the group define hydrophobicity using octanol–water partition coefficients (Log *P*_oct_) that are normalized by the solvent accessible surface area of a macromolecule. These surface-area normalized coefficients are referred to as the Mathers hydrophobicity parameter (MHP, Log *P*_oct_/SA) and have been found to improve the predictive accuracy of polymer hydrophobicity in addition to facilitating comparisons of polymers with architectural differences such as monomer functionality and chain length. A predicted hydrophobicity ranking of our selected homopolymers is therefore summarized in Figure 1b and has been based on a direct or relative comparison of the MHP values determined for a range of example polymers.^43^ Importantly, in their assessment of amphiphilic diblock copolymers which consisted of *N,N*-dimethyl acrylamide (DMA) and various alkyl acrylates such as butyl acrylate (BA), it was also determined that Log *P*_oct_/SA increased significantly with the hydrophobic mol % in the copolymer.^44^ For example, at a hydrophobic monomer mol % ratio of 10, the calculated MHP value of a p(BA)-*b*-p(DMA) diblock copolymer was approximately 0.00074 Å^–2^ in comparison to 0.0027 Å^–2^ at a hydrophobic monomer mol % ratio of 20.^44^

It should be noted that the predictive hydrophobicity values do not account for the influence of salts applied in our study (NaCl, 0.1 M); see Section 4.3.4. At certain ionic strengths, the presence of NaCl can decrease the solubility of nonpolar compounds in water. However, it has generally been observed that the relevance of this effect is minor at physiological concentrations (Na^+^: 0.103 M and Cl^–^: 0.142 M).^45−47^ Similarly, in this work, empirical comparisons of the studied conjugates in salt containing (0.1 M) and salt-free aqueous solutions showed no distinctive differences in their solubility behavior. Furthermore, the salt concentrations applied were found to not substantially influence the self-assembly behavior of the conjugates, and this is indicated by the similarities in the distribution profiles collected using a salt-free eluent; see Figure S6 and Table S1.

### 2.2 Influence of Amphiphilic Polymers

**Figure 2:**
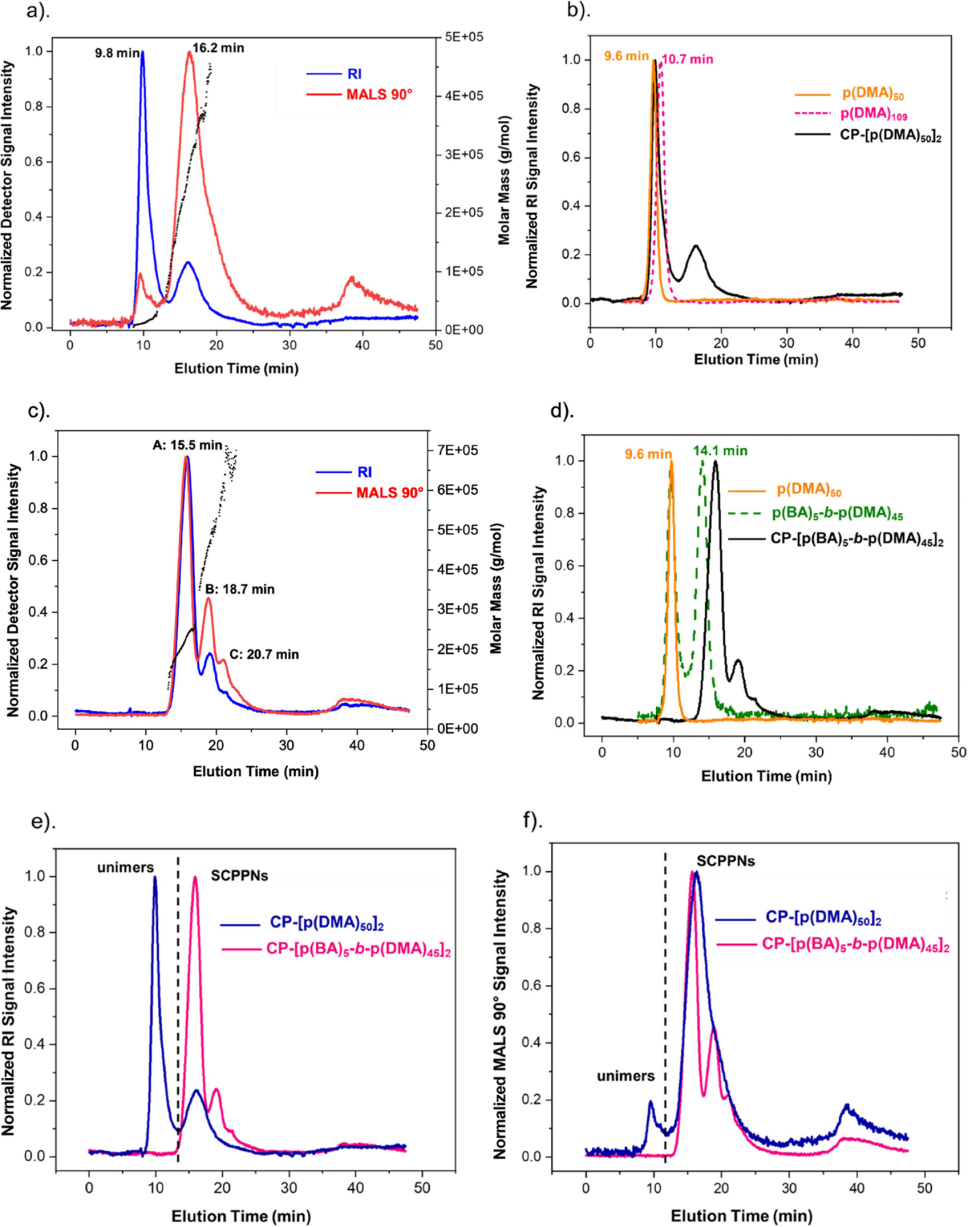
(a) AF_4_-MALS-RI fractograms of the hydrophilic conjugate (**1**, CP-[p(DMA)_50_]_2_) with the molecular weight values for each elution fraction overlaid as black triangles (right *y*-axis). (b) AF_4_-RI fractograms comparing conjugate (**1**) to nonassembling, hydrophilic polymer controls. (c). AF_4_-MALS-RI fractograms of amphiphilic conjugate (**2**, CP-[p(BA)_5_-*b*-p(DMA)_45_]_2_) as an example; see Supporting Information for other amphiphilic systems. (d) AF_4_-RI fractograms comparing conjugate (**2**) to unconjugated hydrophilic and amphiphilic polymer controls to rule out the presence of micelles and free unimers. (e) AF-RI and (f) AF-MALS (90° scattering angle) signal comparison of conjugates (**1**) and (**2**).

We initially evaluated a hydrophilic conjugate (**1,** CP-[p(DMA)_50_]_2_) with poly(dimethyl acrylamide) arms against amphiphilic conjugates containing butyl acrylate blocks (**2**-**4,** CP-[p(BA)_5,10,15_-*b*-(pDMA)_45,40,35_]_2_), in order to interpret the structural distribution in context of our past FRET research where kinetic differences in the unimer exchange behavior were noted.^14,19^ As anticipated, differences are also reflected in the collected fractograms, most notably from the number of detected populations (modality) and their relative abundance (Figures 2 and S1a,b).

The fractogram of the hydrophilic conjugate indicates the presence of two populations (Figure 2a). The earlier eluting population at 9.8 min aligns with unimeric species, as its signal overlaps with those from unconjugated hydrophilic polymers (pDMA_50_ and pDMA_109_, Figure 2b). These polymers were synthesized as standard references for one- and two-arm conjugate unimers since they are similar in molecular weight and do not self-assemble. Importantly, they are also distinguishable from the conjugates based on their UV–vis absorption maxima (see the Supporting Information).

With respect to the second population, its higher MALS signal intensity and later elution time indicate the presence of species larger than the unimers hence are related to self-assembled structures.^25,48^ For each population, the number of building blocks that make up the contained structures, i.e., the aggregation number (*N*_agg_, Table 1), is consistent with the peak assignments. The assignments are further corroborated by structural studies via small angle neutron scattering (SANS). Figure S7a shows the SANS scattering profile of the conjugate which is best modeled using a combined Gaussian coil and core–shell cylinder form factor model. This model is representative of the presence of both unimers and assembled structures of a tubular morphology. The skewing of the scattering curve toward the smaller Gaussian coil structures in the high-*q* region is also in agreement with the relative concentrations calculated for the populations detected by AF_4_ (Table 1). The latter results show that the unimers are substantially more concentrated in solution (64%) than the nanotube assemblies (36%). This can be correlated to a high unimer dissociation behavior which further confirms the conjugate’s highly dynamic nature when taken in conjunction with the fast unimer exchange rate recorded for this system.^14,19^

**Table 1:** Summary of the Relative Concentrations and Size Parameters (Molecular Weight Averages (*M*_w_, *M*_n_*)*, and Aggregation Number (*N*_agg_)) of Each Detected Population as Determined From the Detector Signals^a^

conjugate	population^b^	% relative conc	*M*_w_^c^	*M*_n_^c^	*N*_agg,_ range^d^	2 RSD (%)
**1,** CP-[p(DMA)_50_]_2_	9.8 min	64.39	1.383 × 10^4^	1.238 × 10^4^	N/A ← **1** → 2	+ 100
	A: 16.2 min	35.61	2.446 × 10^5^	2.066 × 10^5^	13 ← **21** → 31	+47.6,–38.1
**2,** CP-[p(BA)_5_-*b*-p(DMA)_45_]_2_	A: 15.5 min	78.84	2.166 × 10^5^	2.131 × 10^5^	16 ← **18** → 21	+16.7,–11.1
	B: 18.7 min	16.48	4.327 × 10^5^	4.279 × 10^5^	33 ← **37** → 42	+13.5,–10.8
	C: 20.7 min	4.67	6.198 × 10^5^	6.150 × 10^5^	50 ← **53** → 57	+7.6,–5.7
**3,** CP-[p(BA)_10_-*b*-p(DMA)_40_]_2_	A: 17.1 min	74.94	4.182 × 10^5^	3.933 × 10^5^	26 ← **35** → 48	+37.1,–25.7
	B: 21.1 min	18.23	9.494 × 10^5^	9.325 × 10^5^	67 ← **79** → 96	+21.5,–15.2
	C: 23.8 min	6.83	1.638 × 10^6^	1.617 × 10^6^	121 ← **136** → 160	+17.7,–11.0
**4,** CP-[p(BA)_15_-*b*-p(DMA)_35_]_2_	A: 19.1 min	56.09	6.110 × 10^5^	5.638 × 10^5^	35 ← **50** → 71	+42.0,–30.0
	B: 23.4 min	21.37	1.422 × 10^6^	1.387 × 10^6^	95 ← **116** → 145	+25.0,–18.1
	C: 31.7 min	22.54	3.626 × 10^6^	3.208 × 10^6^	189 ← **295** → 434	+47.1,–35.9
**5,** CP-[p(HA)_5_-*b*-p(DMA)_45_]_2_	A: 15.9 min	74.58	2.610 × 10^5^	2.445 × 10^5^	16 ← **22** → 29	+31.8,–27.3
	B: 18.6 min	20.67	5.317 × 10^5^	5.220 × 10^5^	38 ← **44** → 53	+20.5,–13.6
	C: 20.7 min	5.97	8.081 × 10^5^	8.047 × 10^5^	63 ← **67** → 73	+9.0,–6.0
**8,** CP-[p(S)_5_-*b*-p(DMA)_45_]_2_	A: 16.1 min	75.98	2.246 × 10^5^	2.165 × 10^5^	16 ← **20** → 25	+25.0,–20.0
	B: 19.3 min	18.26	4.719 × 10^5^	4.650 × 10^5^	35 ← **41** → 49	+19.5,–14.6
	C: 21.7 min	5.76	7.742 × 10^5^	7.701 × 10^5^	62 ← **67** → 74	+10.5,–7.5

a The tabulated values are the means of three data sets/repeats and have low standard errors; see the Supporting Information.

b Populations peaking at the specified elution times, alphabetized populations refer to nanotube assemblies.

c Common units: Da or g mol^–1^.

d The size range (*N*_agg_= *M*_w(assembly)_ ÷ *M*_w(unimer)_) of the assemblies within the specified peak. The range is calculated from the weight-average molecular weight and the upper and lower molecular weight limits where 75% of the peak distribution lies within ±2 standard deviations (2σ) away from the mean. The limits were derived from the cumulative molecular weight distribution plots of each peak. RSD is the standard deviation on a relative scale: (σ/mean) × 100%.

In contrast to the hydrophilic conjugate, a trimodal peak distribution was recorded for all three butyl acrylate conjugates (Figures 2c and S1a,b). By comparing the detected populations to the relevant polymer controls (Figures 2d and S2), we confirm that there are no unimers in solution within detection limits. Furthermore, the comparisons show that the detected populations resulted from the self-assembly of the conjugates and were not due to the aggregation of unconjugated amphiphilic diblock copolymers. The obtained results once again align well with SANS structural analyses whereby the scattering profiles could not be modeled to unimeric species. Statistically reliable fits could however be obtained using a core–shell cylinder form factor, hence suggesting that the structures formed by the conjugates predominantly correlate to nanotubular assemblies (Figure S7a). It is thus apparent that unimer dissociation is significantly minimized by incorporating a hydrophobic segment and this can be attributed to the shielding of the core from the hydrogen-bond competitive water molecules.^14^

The collective results also crucially reveal that the rate of unimer dissociation influences the abundance of the assemblies. This is well illustrated by a fractogram comparison of the hydrophilic (**1,** CP**-**[ p(DMA)_50_]_2_) and amphiphilic conjugates (**2,** CP**-**[p(BA)_5_-*b*-p(DMA)_45_]_2_), whereby significant concentration differences are highlighted between assemblies of similar sizes formed by both conjugates (Figure 2e,f). In particular, the hydrophilic assemblies are seen to be considerably lower in concentration which can be associated with their higher predisposition to revert back into unimers due to solvent interference of their intermolecular H-bonding. Conversely, it is hypothesized that the amphiphilic assemblies are more concentrated because they can maintain their self-assembled state as a consequence of the reduced unimer dissociation. These findings also contextualises previously published light scattering data where it was postulated that the hydrophilic conjugate assembled into considerably shorter nanotubes than those formed by an amphiphilic system.^14^ However, since the calculated size values were the averages of unfractionated distributions, it is plausible that those determined for the hydrophilic system were underestimated by the high unimer population observed in this study.

### 2.3 Effect of Gross Hydrophobic Quantity

#### 2.3.1 Hydrophobic–Hydrophilic Block Length Ratio

Having shown that the implementation of a hydrophobic block significantly inhibits the dissociation of unimers, we next sought to further elucidate the relevance of increasing the gross hydrophobic content. This was first examined using conjugates (**2**–**4**) that represent a sequential increase in the hydrophobic (pBA) to hydrophilic (pDMA) block ratio. These conjugates, as discussed in Section 2.2, contain three detected populations which are consistent with nanotube structures that are referred to in the text as populations A, B, and C respective to their ascending elution time (Figures 2c, S1a,b and S7a).

**Scheme 1:**
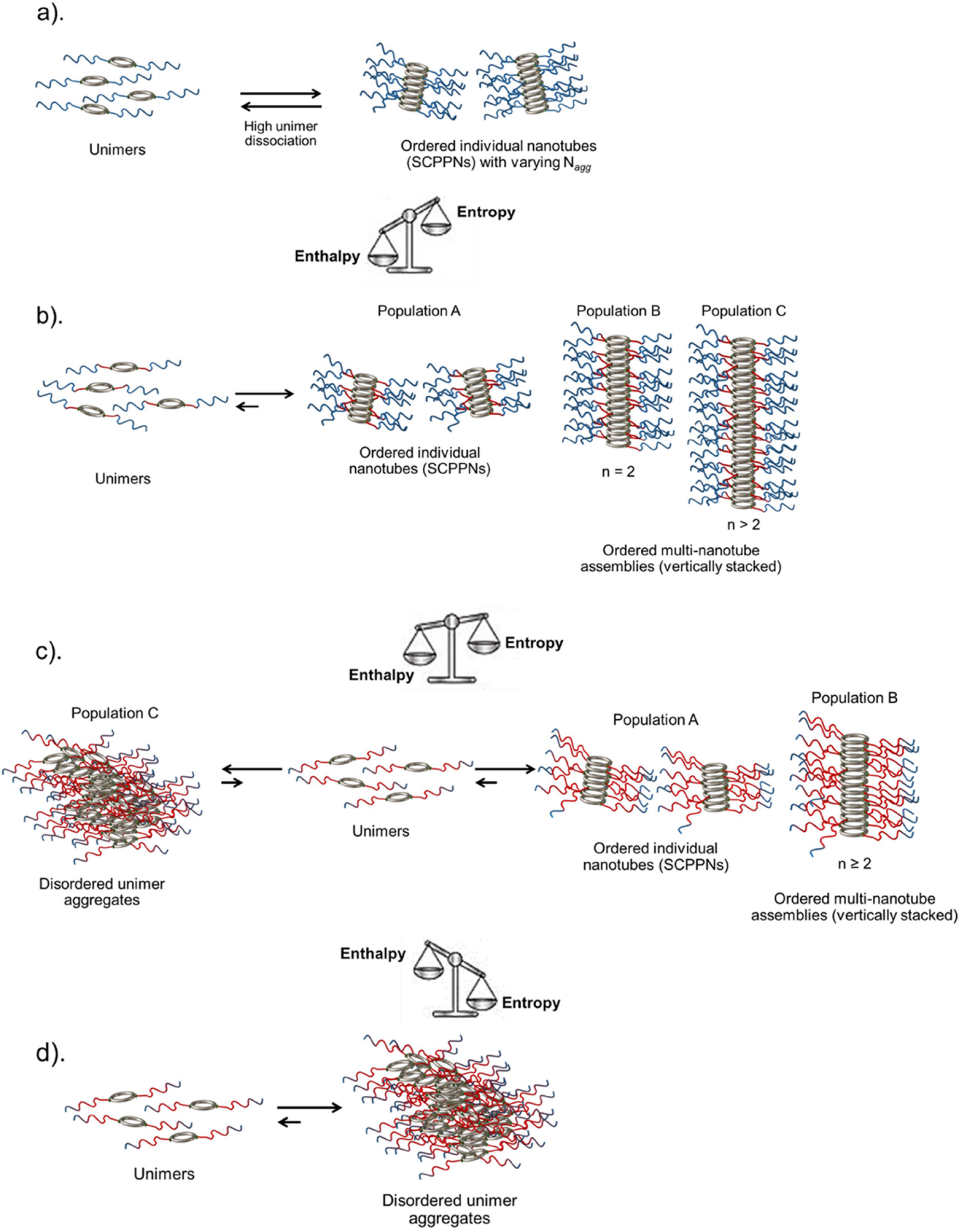
Schematics Illustrating the Hypothesized Association Behaviour of the Studied Conjugates (a) The self-assembly of the hydrophilic conjugate (**1**), characterized by high unimer dissociation. (b) The self-assembly of the amphiphilic conjugates with a low hydrophobic content, conjugates (**2**, **3**, **5**, **8**). The self-assembly is governed by the enthalpically favorable formation of an extensive H-bond network. (c) The amphiphilic conjugates (**4**, **6**) whose self-assembly via H-bonding is retarded by the entropically driven hydrophobic effect that is characterized by the disordered aggregation of unimers. (d) The amphiphilic conjugates (**7, 9, 10**) whose association is predominantly governed by the hydrophobic effect. NB: n = average number of associating individual nanotubes. The polymer hydrophilicity and hydrophobicity is represented in blue and red, respectively.

Interestingly, the light scattering data of conjugates (**2**, CP-[p(BA)_5_-*b*-p(DMA)_45_]_2_) and (**3**, CP-[p(BA)_10_-*b*-p(DMA)_40_]_2_) indicate that the later eluting assemblies (populations B and C) successively increase or double by the size of nanotubes in population A, thus implying that some association among the nanotubes in population A had occurred (Table 1). Taking into account the SANS structural analysis, in addition to the steric hindrance expected from the conjugates’ hydrophilic polymer shells, it is hypothesized that the larger nanotube assemblies did not result from lateral aggregation.^38^ We instead argue that these structures were formed due to the vertical stacking of the nanotubes in population A since this behavior has previously been observed. In particular, STORM images of model amphiphilic conjugates designed with Cy3 and Cy5 dye pairs clearly distinguished that single nanotubes resulting from these conjugates could combine at their termini to form larger segmented nanotube structures.^14^ Using this assumption, population A is thus defined as a distribution of single/individual nanotubes (SCPPNs) whereas the structures in populations B and C are described as multinanotube stacks, see Scheme 1.

**Figure 3:**
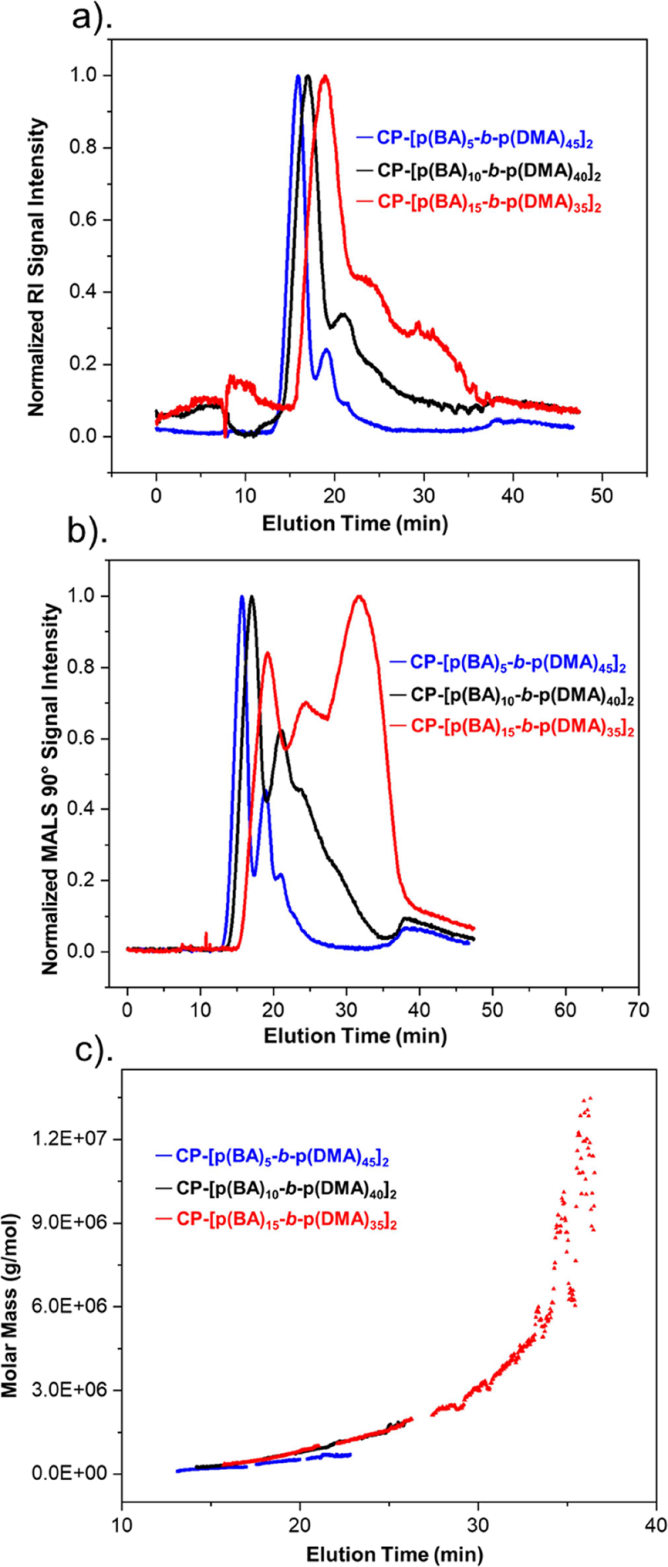
Comparison of the amphiphilic butyl acrylate conjugates by their hydrophobic block ratio (10–30 mol %). (a) AF-RI signals, (b) AF-MALS (90° scattering angle) signals, and (c) molecular weight profiles.

By comparison of the elution times (Figure 3a–c) and molecular weight profiles of the nanotube assemblies from the two conjugates, it is apparent that their sizes are directly proportional to the hydrophobic block ratio. This suggests that the hydrophobic content introduced next to the CP core influences the degree of association between unimers. Differences in the degree of association are clearly marked by the calculated molecular weight averages and *N*_agg_ values of the single nanotubes, which show that conjugate (**3**) forms twice as large nanotube structures as conjugate (**2**), see Table 1. The multinanotube assemblies within population C also highlight differences in the degree of association as they represent the highest observed number of individual nanotubes interacting with each other on average. Results show that, on average, up to 4 single nanotubes formed by conjugate (**3**) could associate with each other in comparison to conjugate (**2**) whose average is 3 nanotubes. Furthermore, by estimating the dispersity of the nanotube assemblies based on the 75% empirical rule, the differences in the degree of association could be validated.^49,50^ This rule describes the limits where at least 75% of the values of any distribution fall within 2 standard deviations (2σ) away from the mean, hence serves as a useful measure of the dispersity.^49−51^ According to our calculations, increasing the hydrophobicity from 10 to 20 mol % significantly increases the size limit that unimeric species can self-assemble up to (Table 1, **+RSD**). A similar trend is noted with the multinanotube assemblies showing that increasing the hydrophobic content increases the association between the SCPPNs, but to a smaller relative scale. These results, in combination with our earlier discussion (Section 2.2), thus reveal a synergistic influence of incorporating a hydrophobic inner layer. Precisely, we propose that the dissociation of the CP-unimers is restricted while the H-bonding between them is simultaneously enhanced, hence the increased association.

These observations can be argued against two theories accounting for the influence of the hydrophobic blocks on the solvation of the conjugates and/or their shielding of the intersubunit hydrogen bond network. Briefly, in the former case, it is worth noting that aqueous solvation of hydrophobic molecules at room temperature is generally considered to be dominated by entropic changes to the solvent.^52−56^ In particular, solvation in water is characterized by a decrease in the entropy of the water molecules since they arrange themselves as ordered clathrate cages. This decrease is proportional to the hydrophobic surface area and may be accompanied by enthalpic contributions of the same order of magnitude.^53,57^ In this regard, it is logical that as the hydrophobic quantity is sequentially increased to 20 mol %, the self-assembly of unimers via enthalpically driven hydrogen-bonding becomes more favorable. This results in a compensation phenomenon since stacking confines the hydrophobic segments, which in turn increases solvent entropy by liberating the ordered water molecules.

This increase in the enthalpic attraction between the subunits can be correlated to the role of the inner hydrophobic polymer layers in shielding the backbone hydrogen bonding sites from competitive water molecules. In their study on the binding between mannoside ligands and lectin, Cramer et al. reported a −13 kJ mol^–1^ enthalpic contribution that was a direct consequence of H-bond shielding.^58^ In contrast, it was found that when the hydrogen bond network in the binding site was disrupted, the enthalpic benefit was completely abolished. Shielding of H-bonds by hydrophobic entities has also been reported to strengthen the H-bonds as they provide low dielectric environments in which dipole–dipole “electrostatic” interactions are stabilized.^58−62^ Importantly, simulation research by Barril and co-workers suggested that the level of shielding, and hence the influence on enthalpic contributions, is likely proportional to the hydrophobic quantity.^60^

The discussed shielding influence on enthalpic contributions may also explain the formation of the multinanotube stacks. Specifically, it is posited that by increasing the enthalpic association between subunits, the free energy penalties imposed by the polymer steric bulk on CP-association is more readily overcome.^41,42^ Alternatively, with respect to solvent entropy compensation, the formation of the multinanotube stacks may occur to increasingly minimize the interfacial surface area between the nonpolar groups and the water molecules.^56^ Remarkably, for both systems, the relative abundance of the individual nanotubes (≥75%, Table 3) is considerably higher than the multinanotube stacks following equilibration for 24 h. These concentration differences are elucidated in a separate kinetic study monitoring the evolution of the structures formed by conjugate (**2**), *manuscript in preparation*. The results indicated that self-assembly precedes with the formation of single nanotubes which gradually convert into the multinanotube structures observed in population C. Moreover, it was observed that the structures contained in population C did not reconfigure over the long time period between their formation and the last measured time point (between 12 and 24 h). This therefore implies that the populations A and B are metastable configurations whose conversion into the forward assemblies is associated with activation energy barriers.^63^

The results collected for conjugate (**4**, CP-[p(BA)_15_-*b*-p(DMA)_35_]_2_) further establish that increasing the hydrophobic block length ratio increases association. Notably, by comparing the butyl acrylate fractograms, the presence of disproportionately larger structures is observed in the distribution of conjugate (**4**) at 31.7 min (Figure 3b,c and Table 1). This is supported by its SANS scattering profile as the formation of larger structures is indicated at the low *q*-regime (*q* = 0.01) where the slope scales to a higher intensity instead of leveling off (Figure S7a). According to the SANS data fitting and our assumption of vertical stacking, these structures may correspond to significantly larger multinanotube stacks; however, precipitated aggregates were also noticed in solution which could signify that the structures resulted from disordered aggregation driven by the hydrophobic effect (Figure S4a). Briefly, this behavior is postulated to be characterized by a less controlled aggregation behavior, whereby the unimers randomly associate in a manner that buries the hydrophobic cavities away from the solvent (Scheme 1). This less restricted behavior is consistent with the formation of large aggregates which readily precipitate and would also account for the polydispersity discrepancies noted for these structures in comparison to the assemblies formed by conjugates (**2**) and (**3**). In particular, the results show that the presumed ordered stacking of the individual nanotubes from conjugates (**2**) and (**3**) sequentially decreases the polydispersity of the resulting multinanotube stacks (**±RSD,**Table 1). In contrast, the polydispersity of the largest structures from conjugate (**4**) opposingly increases, which implies that they may have not resulted from the ordered system behavior.

An appreciable difference is also apparent with regard to the relative abundance (21%) of the large structures formed by conjugate (**4**), in comparison to the largest assemblies of the other butyl acrylate conjugates (5–7%). This indicates that a greater proportion of the unimers from the conjugate (**4**) was driven to associate into the highest possible size limit. Separate conclusions can be drawn by interpreting these differences against the conjugate design and the proposed system behavior mechanisms of ordered stacking vs disordered aggregation. First, it can be rationalized that the conjugate design imposed a much higher solvent entropic cost due to the increased hydrophobic surface area and the lower solvation contributions from the shortened hydrophilic shell. Consequently, in accordance with the earlier discussion, it can be argued that the enthalpic association between unimers was substantially augmented thereby driving the system to form larger nanotubes via ordered vertical stacking. Alternatively, it is plausible that the further increase in the solvent entropic penalty resulted in a behavior whereby the enthalpic benefit of β-sheet stacking is significantly counteracted by the hydrophobic effect.^56^ This could be because compensation by the hydrophobic effect would liberate ordered water molecules without greatly reducing the system entropy (i.e., Δ*S*_system_ for disordered aggregates is less negative than Δ*S*_system_ for ordered nanotubes).^64,65^ It is also probable that the formation of the disordered aggregates is characterized by lower activation energy barriers in comparison to the formation of nanotubes whose growth mechanism is generally accepted as cooperative (unfavorable nucleation).^27,40,56,66−69^

As a final point, it is also worth noting that the role played by the amphiphilic polymers in reducing unimer dissociation and driving association is a key contributing factor to the resulting assembly polydispersity (Table 1). For example, the individual nanotubes formed by hydrophilic conjugate (**1**) exhibit the highest dispersity. This dispersity is expected since their high unimer dissociation gives rise to a stochastic array of nanotubes with fluctuating *N*_agg_, as demonstrated by their broad continuous peak width (Figure 2a). The attachment of amphiphilic polymers helps reduce the dispersity caused by unimer dissociation, as verified by the lower standard deviations obtained for the amphiphilic assemblies. This is however most notable with conjugate (**2**) which has a small hydrophobic block ratio (10 mol %), therefore indicating that as association proportionally increases with hydrophobicity, so will the dispersity. This is intuitive since extending the growth limit of the conjugates systems will increase the probability of forming a wider size range of assemblies. The polydispersity is also significantly affected by the manner in which association occurs (ordered vs disordered), as implied by the conjugate (**4**).

#### 2.3.2 Hydrophobic Monomers

For the second part of our analysis, we investigated whether the influence of increasing the hydrophobic content could be better tuned by changing the hydrophobic monomer. Additional conjugates comprised 10 mol % hydrophobic blocks of hexyl acrylate (**5,** CP-[p(HA)_5_-*b*-p(DMA)_45_]_2_) and styrene (**8,** CP-[p(S)_5_-*b*-p(DMA)_45_]_2_) monomers were therefore studied against the equivalent butyl acrylate conjugate (**2**). We anticipated that using more hydrophobic monomers would offer an efficient way of enhancing self-assembly while minimizing the risk of disordered behavior. The latter was theorized to occur when the solvation contributions of the short hydrophilic arms are dominated by an increased hydrophobic surface area; see conjugate (**4**).

**Figure 4:**
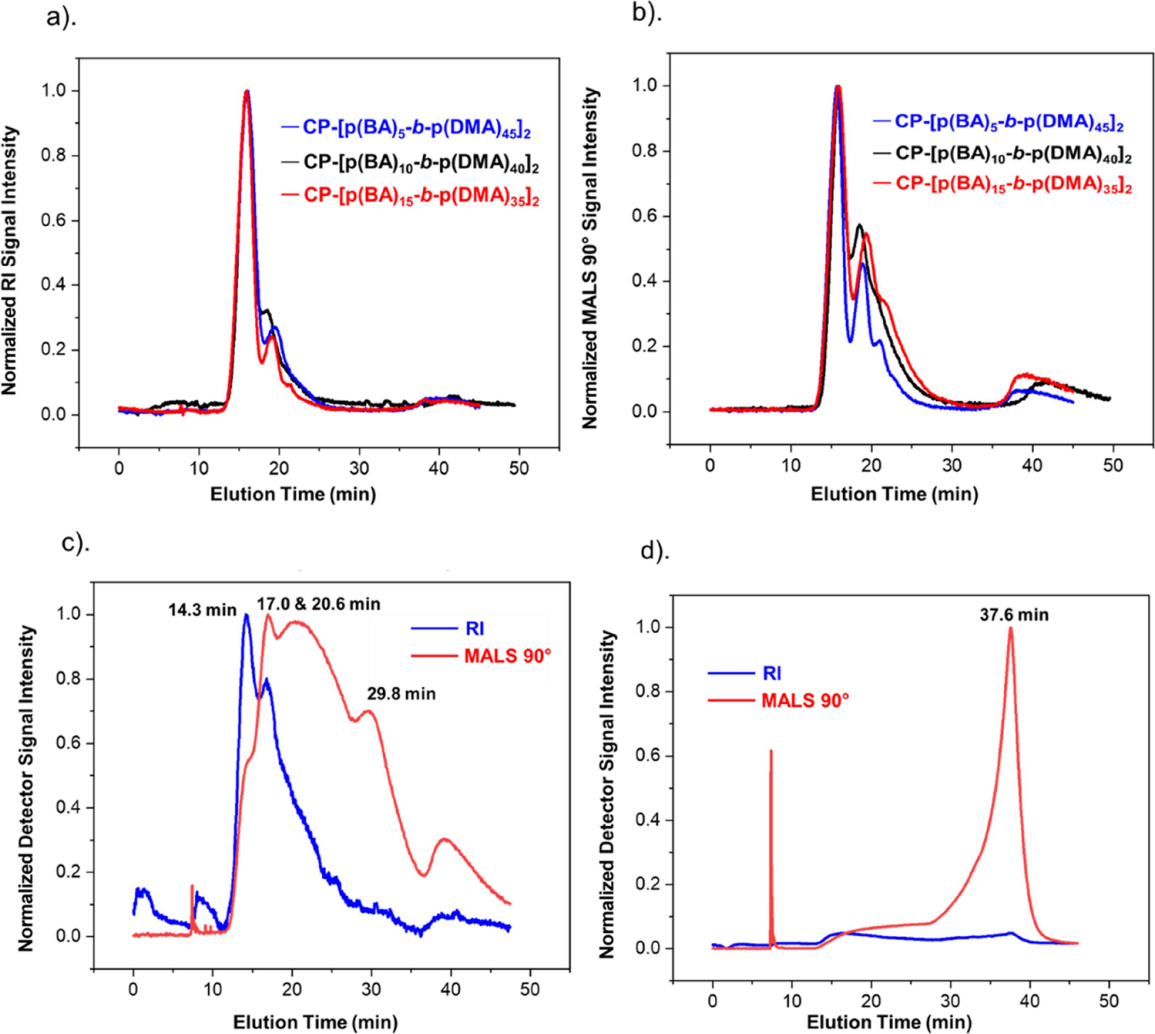
(a, b) Comparison of the amphiphilic conjugates by their hydrophobic monomers at 10 mol % hydrophobicity. (c) AF_4_-MALS-RI fractogram of conjugate (**6**, CP-[p(HA)_10_-*b*-p(DMA)_40_]_2_). (d) Example AF_4_-MALS-RI fractogram of the insoluble conjugates (**7**, CP-[p(HA)_15_-*b*-p(DMA)_35_]_2_), (**9**, CP-[p(S)_10_-*b*-p(DMA)_40_]_2_) and (**10**, CP-[p(S)_15_-*b*-p(DMA)_35_]_2_).

Surprisingly, negligible differences are marked from a visual analysis of the collected fractograms and SANS scattering profiles of the conjugates. The fractograms show that the conjugates have trimodal distributions where the detected populations elute in close proximity, thus indicating that the conjugates form assemblies that are homogeneous in size (Figure 4a,b, Table 1). A cylindrical architecture is determined for these assemblies from the fitting analysis of the conjugates’ SANS profiles, and the similarity of their sizes is supported by their scattering intensities which are within the same scale (Figure S7b). In addition, the molecular weight averages calculated for the assemblies of each conjugate show a matching trend with regard to their self-assembly behavior which has been discussed above in Section 2.3.1, see also Scheme 1. Some distinction in the degree of association is indicated from the dispersity results (Table 1), whereby it is noted that the hexyl acrylate and styrene conjugates can self-assemble to higher size limits. Nonetheless, these differences do not appear to have an appreciable effect on the averaged population profiles. We therefore designed another series of conjugates with higher hydrophobic block ratios in order to further evaluate the influence of the hydrophobic monomer.

Increasing the hydrophobic block ratio to 20 mol % drastically impacted the solubility of the conjugates comprising more hydrophobic monomers. For instance, a colloidal solution was formed by conjugate (**6,** CP-[p(HA)_10_-*b*-p(DMA)_40_]_2_), demonstrating poor solubility hence the likely occurrence of uncontrolled aggregation (Figure S4b). The latter hypothesis is consistent with the conjugate’s measured fractogram which shows the presence of nondiscrete populations within a multimodal distribution (Figure 4c). A qualitative approach was employed to assign the detected peaks since the concentration and molecular weight averages could not be calculated. This is because erratic RI signal responses were measured when estimating the conjugate’s refractive index increment (∂*n*∂*c*); see Section 4.3.7. Comparing the conjugate’s fractogram with polymer controls confirms the lack of free unimers and that the populations relate to the conjugate system (Figure S2). Moreover, the presence of nanotube structures is supported by the core–shell cylinder model applied to the SANS data (Figure S7c), but notably, the strong aggregation visible in solution is not conclusively indicated across the accessible low-*q* range as a steep upturn of the scattering intensity should have been observed. Further conclusions were subsequently inferred by relating the conjugate’s populations to those formed by the other systems. First, the population at 17 min is postulated to be a distribution of ordered nanotube assemblies since its signal overlaps with the peak related to single nanotubes from conjugate (**3**), see Figures S3 and Table 1. The peak eluting at 14.3 min is therefore also assumed to relate to nanotube structures but of a smaller size as indicated by its lower MALS signal intensity.^25^ It is likely, but inconclusive, that the smaller nanotube structures are the single nanotubes which vertically stack to form the multinanotube assemblies at 17 min. In contrast, the larger populations at 20.6 and 29.8 min are attributed to the disordered aggregation of unimers (Scheme 1). This is corroborated by their weak and low signal-to-noise ratio of their concentration signals, which combined with the precipitation observed, suggests that the structures are poorly soluble. Furthermore, the very broad distribution widths of these populations imply a high polydispersity, which is in contrast to the polydispersity trend observed from the assemblies presumed to form due to ordered vertical stacking, specifically those formed by conjugates (**2**), (**3**), (**5**), and (**8**). As discussed earlier, this trend suggests that the polydispersity decreases as the systems self-assemble into ordered multinanotube structures (Table 1, **±RSD**). Based on these assignments, it is interesting to note that the nanotube assemblies of this conjugate are smaller than those formed by its equivalent butyl acrylate conjugate (**3**), see Figures S3. We argue that this is because of the competition from the entropically driven association behavior which would retard the self-assembly of unimers into ordered assemblies.

The styrene conjugate, (**9**, CP-[p(S)_10_-*b*-p(DMA)_40_]_2_), demonstrated even lower solubility as it heavily precipitated into a cloudy suspension that gradually sedimented (Figure S4c,d). Its collected fractogram shows no RI signal and a fronting MALS peak at 37.6 min which could not be quantitatively analyzed due to the lack of a measurable RI signal for ∂*n*∂*c* determination (Figure 4d). Regardless, when interpreted alongside the employed crossflow field programming, see Section 4.3.5, the observed detector signals suggest the formation of large insoluble structures, as they would be undetectable by the RI detector and would only elute at the end when the retention force field was at its minimum (0.07 mL/min). Therefore, based on the observations taken from the fractogram and solution, it is reasonable to assume that the conjugate predominantly exhibits a disordered association behavior driven by the hydrophobic effect (Scheme 1). Similar conclusions were drawn regarding the use of the more hydrophobic monomers at a hydrophobic block ratio of 30 mol %, conjugates (**7**, CP-[p(HA)_15_-*b*-p(DMA)_35_]_2_) and (**10**, CP-[p(S)_15_-*b*-p(DMA)_35_]_2_). At this block ratio, the precipitation of the conjugates intensified, and the resulting fractograms were equivalent to that observed with conjugate (**9**). It is therefore evident that the influence imposed on the system behavior relies on a subtle interplay between the hydrophobic monomer and the hydrophobic to hydrophilic block length ratios. Moreover, the observed solubility and polydispersity trends strengthen our hypothesis that a disproportionate increase in the hydrophobic quantity deviates the systems from an ordered self-assembly behavior.

In summary, we hypothesize that the inclusion of a hydrophobic inner layer drives the conjugate systems to form progressively larger assemblies in order to compensate the solvent entropic penalty. It is postulated that at low hydrophobicity, this penalty can be readily compensated by the formation of ordered multinanotube assemblies which arise from an enthalpically driven β-sheet stacking behavior of conjugate units. However, when a high hydrophobic quantity is not proportionately balanced against the solvation properties of the hydrophilic polymer block, the enthalpic contributions of the H-bonding behavior will not favorably resolve the greater requirement to confine the hydrophobic segments. We posit that this is due to the kinetic or energetic constraints associated with the formation of vertically stacked assemblies. This will in turn lead to a gradual shift toward an entropy driven association behavior, which is triggered by the hydrophobic effect and is characterized by the less ordered aggregation of the unimers.^50,56^

## 3 Conclusion

In this work, the influence of amphiphilic diblock copolymers on the self-assembly behavior of supramolecular α-alt(d, l)-cyclic peptide systems has been systematically evaluated via asymmetric-flow field flow fractionation and small angle neutron scattering.

A high unimer dissociation behavior is demonstrated for CP systems conjugated with hydrophilic homopolymers, and this significantly affects the stability of their resulting nanotube assemblies. This is confirmed by comparing the distributions of these systems against those conjugated with amphiphilic diblock copolymers. The distribution of the hydrophilic system highlights a high concentration of free unimers in comparison to those of the nanotube assemblies. In contrast, no unimers are detected for the amphiphilic systems, and the abundance of their nanotube assemblies is notably higher than those formed by the hydrophilic conjugate. These results are related to the level of solvent interference experienced by the conjugates. In particular, the amphiphilic conjugates are designed to have a shielding hydrophobic polymer block adjacent to the CP core; hence, solvent molecules competing for the hydrogen bonding sites between peptides are excluded. This consequently limits the unimer dissociation and allows the nanotube assemblies to maintain their self-assembled state. Conversely, hydrophilic systems are susceptible to the disruption of their intermolecular H-bonds, and thus, nanotube assemblies readily dissociate back to unimers.

In addition to preventing unimer dissociation, the results indicate that the incorporation of a hydrophobic block influences the association between conjugate units. The influence is dependent on the hydrophobic block’s molecular design as this determines the quantity of hydrophobicity introduced. This has been systematically evaluated by adjusting the hydrophobic block ratios or the hydrophobic monomer of the amphiphilic diblock copolymers. A low hydrophobic quantity has been found to synergistically enhance the H-bonding association of conjugates, thus resulting in the formation of elongated multinanotube assemblies. It is theorized that this behavior compensates the entropic penalty of ordering water molecules around the hydrophobic blocks of free unimers. However, this effect relies on a delicate relationship between the hydrophobic and hydrophilic block ratios as well as the hydrophobic monomer. Consequently, when the hydrophobic content is disproportionately increased, it is hypothesized that the thermodynamics regulating the self-assembly process is altered. In this case, the β-sheet stacking of conjugates is significantly counteracted by the hydrophobic effect. According to our theory, this will provide a less energetically restricted pathway for the conjugates to associate and lower the hydrophobic interfacial area exposed to the solvent. This entropically driven association is characterized by the random aggregation of the unimers, which leads to the formation of very large aggregates that readily precipitate.

Lastly, our study establishes a dispersity pattern that correlates to not only the extent of unimer dissociation but also the association behavior. With regard to the ordered nanotube assemblies, the highest dispersity is recorded for the hydrophilic assemblies. This is attributed to their high unimer dissociation, which enables them to stochastically disassemble into varied sizes. The attachment of amphiphilic polymers helps reduce the dispersity caused by unimer dissociation, but this is dependent on the hydrophobic quantity. The polydispersity proportionally increases with hydrophobicity as this increases the H-bonding association and thus extends the growth limit of the conjugates systems. The manner in which the assemblies are formed (ordered vs disordered) also influences the polydispersity. The disordered aggregation of unimers results in a significant increase in the polydispersity in comparison to the ordered stacking of nanotube assemblies, and this has been used to distinguish the assembly types.

In summary, it is anticipated that the data presented here serve as a guide on optimizing the design of supramolecular systems to achieve structural control and stability. This is useful for the development of biotechnological applications such as drug delivery where the control over the size, dispersity, and stability of the vectors is often relevant.^50,70−72^ From a synthetic standpoint, our findings also lay the basis for further investigation of the influence of the hydrophobic content. A key interest for the future is understanding whether the dispersity within the hydrophobic blocks plays a major role, as observed with the micellar self-assembly of amphiphilic diblock copolymers.^50^ Finally, the implementation of field flow fractionation has been demonstrated to be an efficient technique for measuring the distribution of supramolecular systems. The availability of this information has been an important factor in understanding the mechanistic impact of the conjugate designs in addition to validating our previous research on the dynamics of the systems.

## 4 Experimental Section

### 4.1 Reagents

### 4.2 Conjugate Synthesis

#### 4.2.1 Cyclic Peptide Synthesis

The two arm α-alt(d, l)- cyclic peptide used in this study was synthesized from a linear octapeptide precursor with the amino acid sequence, (d-Leu-l-Trp- d-Leu-l-Lys-)_2_ and a detailed synthesis protocol is available in the referenced literature.^38^ The final cyclic product, as well as its precursors, were characterized by electrospray ionization time-of-flight mass spectrometry (ESI-ToF-MS) and ^1^H NMR spectrometry in order to confirm the identity and purity of the products. See Supporting Information, Section C.

#### 4.2.2 Polymer Synthesis

The hydrophilic polymers and amphiphilic diblock copolymers were synthesized using the group’s well-established RAFT polymerization protocol which is available in the following literature.^14^ Polymer conversions were determined using ^1^H NMR and a high conversion (≥98%) was achieved for all the final and intermediate polymers. Before purification, SEC characterization was performed to ensure that the reactions were well controlled based on the measured distribution and dispersity (*Đ*). SEC characterization of the amphiphilic diblock copolymers was also employed to confirm successful chain extension. Lastly, the purity of the final polymers was assessed using both ^1^H NMR and SEC analysis. See Supporting Information, Section C.

#### 4.2.3 Conjugate Synthesis

The conjugation of the polymers to the cyclic peptide was performed according to the procedures outlined in the referenced paper.^14^ Conjugation and purification efficiency was confirmed via SEC characterization, and the collected results are shown in the Supporting Information, Section C.

### 4.3 AF_4_ Analysis

#### 4.3.1 Instrumentation

All measurements were conducted using the PostNova Analytics AF2000 multiflow system (Malvern, UK) with a PN5300 injection autosampler. To allow for a multidetection setup, the company’s differential refractive index (RI) PN3150 detector and multiangle light scattering (MALS) PN3621 detector were connected online to the system. Light scattering was measured using a 50 mW laser operating at a wavelength of 532 nm (green), and the resulting signals were detected from 21 observation angles ranging between 7 and 164°. A single wavelength UV–vis photodiode array detector (Shimadzu, SPD-M20A) was also integrated online to extend the field of view, i.e., used to simply monitor the samples and not used for data calculations. Sample separation was conducted within a channel (335 mm × 60 mm × 40 mm) containing a trapezoidal polytetrafluoroethylene spacer (PTFE, 350 μm thickness) and a semipermeable regenerated cellulose (RC) membrane with a 10 kDa nominal molecular weight cutoff. It should be noted that the channel and all its components were also provided by PostNova Analytics (Malvern, UK). Data acquisition and processing were controlled through the PostNova AF2000 software version 2105.

#### 4.3.2 Detector Calibration and System Performance

Prior to the measurements, calibration of the detectors was performed according to the manufacturer’s manual to determine the detector constants. Lyophilized bovine serum albumin (BSA, 1 mg mL^–1^) prepared in 0.9% NaCl aqueous solvent was used to calibrate the RI detector. The MALS detector was also calibrated using a BSA solution (5 mg mL^–1^) and the scattering angles were normalized against a polystyrenesulfonate sodium salt solution (PSS, 67 kDa, *Đ* ≤ 1.2, 10 mg mL^–1^) or a latex mixture: 3000 Series NIST certified polystyrene nanosphere particle size standards with nominal diameters (nm) of 23 ± 2 (0.4 mg mL^–1^), 61 ± 4 (0.04 mg mL^–1^), and 122 ± 3 (0.008 mg mL^–1^). PSS normalization was performed for soluble macromolecules and the standard solution was prepared using 0.9% NaCl solvent. Latex normalization was conducted for particles and the mixture was prepared using a 0.2% NovaChem surfactant solution: a mixture of nonionic and ionic detergents. Lastly, the calibration and separation performance of the AF_4_ channel were evaluated by fractionating BSA (1 mg mL^–1^). System performance is confirmed by the good resolution of its monomer (66 kDa), dimer, and trimer peaks, and calibration is validated by obtaining the correct molecular weight values of the peaks.

#### 4.3.3 Sample Measurement

All samples were measured at room temperature in an aqueous solution consisting of 0.1 M sodium chloride (NaCl) and 0.02% sodium azide (NaN_3_) (Table 2). NaN_3_ was added to prevent bacterial contamination while the NaCl was added as a standard AF_4_ practice to minimize nonspecific electrostatic interactions between any charged analytes and the AF_4_ regenerated cellulose membrane which carries a weak negative net charge at neutral pH.^74−76^ This is achieved because of the charge screening effects of the Na^+^ and Cl^–^ ions. An additional benefit of the NaCl salt concentration is that it is representative of physiological cell environments (Na^+^: 0.103 M and Cl^–^: 0.142 M).^45^

**Table 2:** List of Reagents (≥95% Purity) and Supplier Information

	reagent	supplier
solvents	dichloromethane (DCM)	Thermo Fischer Scientific
	dimethyl sulfoxide (DMSO)	
	*N, N-d*imethylformamide (DMF)	
	1,4-dioxane	
	HPLC-grade water	
	deuterated solvents	Sigma-Aldrich
polymer synthesis	monomers	Sigma-Aldrich
	V-601 azo-initiator	Fujifilm Wako Chemicals
	2-[[(butylsulfanyl)-carbonothioyl]sulfanyl] propanoic acid (PABTC)	Synthesized by group as in the referenced protocol.^73^
cyclic peptide synthesis	Fmoc-protected amino acids	Iris Biotech GmbH
	2-chlorotrityl chloride resin	Sigma-Aldrich - Novabiochem
	triisopropylsilane (TIPS)	Sigma-Aldrich
	HCTU	
	*N, N-d*iisopropylethylamine (DIPEA)	
	4-methylmorpholine (NMM)	
	piperidine	
	trifluoroacetic Acid (TFA)	Thermo Fischer Scientific
	HATU	Alfa Aesar
	hexafluoroisopropanol (HFIP)	
	DMMTMM·BF_4_	Acros Organics
AF_4_ analysis	sodium chloride (NaCl)	Thermo Fischer Scientific
	sodium azide (NaN_3_)	Sigma-Aldrich

The aqueous eluent was prepared by dissolving the salts in HPLC grade water with the aid of a stirrer. Before use, the eluent was vacuum-filtered through 0.1 μM aqueous compatible filters (e.g., PTFE) to remove any impurities that would interfere with the light scattering signals, particularly at low angles.^25^

#### 4.3.4 Sample Preparation

One mg mL^–1^ solutions of all the conjugates and their respective polymer controls were prepared by first dissolving the samples in 5% dimethyl sulfoxide (DMSO) and then slowly adding 95% of the aqueous measurement eluent. Equivalent solutions of the polymers were also prepared to estimate the refractive index increment (∂*n*∂*c*) values of the conjugates, which is a required parameter for the estimation of the molecular weight and concentration of the populations (Section 4.3.6).

#### 4.3.5 Method Development

The method conditions used to analyze the samples in this study are summarized below in Table 3.

**Table 3:** Programmed AF_4_ Experimental Conditions Used in This Study^a^

step	conditions
injection + focus	injection volume: 50 μL
	injection time: 6 min
	injection flow: 0.2 mL/min
	cross flow (CF): 1.75 mL/min
	focus flow: 2.05 mL/min
	transition time: 1 min
elution (CF Programming)	step 1 (20 min): CF held constant at 1.75 mL/min
	step 2 (10 min): CF decayed linearly from 1.75 to 0.07 mL/min
	step 3 (10 min): CF held constant at 0.07 mL/min
rinse	before the start of the next run, a brief rinse was performed at a channel (TIP) flow rate of 0.1 mL/min for 30 s.

a Note that the channel outlet flow (detector-flow) rate was held constant at 0.5 mL/min during each analysis.

#### 4.3.6 Determination of the Molecular Weight Averages and Concentration

The molecular weight distribution is defined by a series of volume slices (*i*) that each contain a specific concentration of molecules presumed to be similar in their molecular weights.^25,77−79^ The concentration of the molecules in each slice is determined from the refractive index detector signal according to eq 1, while their molecular weight is determined by using the Zimm formalism equation and based on the assumption that the polydispersity of the molecules is negligible. Briefly, the Zimm formalism relates the measured light scattering signal intensity to the molecular weight and concentration of the molecules, eq 2.^25,77−79^ The data obtained from each slice is then used to calculate the molecular weight averages of the distribution, namely, the weight- (*M*_w_) and number-average (*M*_n_), see eqs 3a and 3b.^25,77−79^
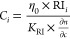
1

RI is the measured refractive index signal, *K*_RI_ is the calibration constant, and η_0_ is the refractive index of the detector cell reference solvent. The subscript *i* indicates the elution volume slice.
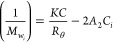
2

*K* is an optical constant that is dependent on the ∂*n*∂*c* of the matrix; *C* is the concentration; *M*_w_ is molecular weight; *R*_θ_ is the excess Rayleigh ratio of the solution (ratio of the scattered and incident light intensity); and *A*_2_ is the second virial coefficient which is a measure of sample–sample interaction.
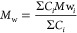
3a
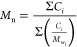
3b

Note that the sum of *C*_*i*_ (∑*C*_*i*_) is proportional to the total concentration under the area of a RI detected peak.

#### 4.3.7 Estimation of the ∂*n*∂*c*

The ∂*n*∂*c* values of the conjugates were estimated online from the RI detector signals of their control polymers; see Supporting Information for justification. Herein, polymer solutions of known concentrations were measured without applying a separation cross-flow field in order to fulfill the assumption of 100% recovery. Any inconsistency in the detected cumulative concentration was therefore related back to an erroneous ∂*n*∂*c* value, and the ∂*n*∂*c* was adjusted until the target concentration was met. At least three consistent measurements were collected per solution and the mean values and associated standard deviations are recorded in Table S2.

#### 4.3.8 Estimation of the Dispersity (Upper and Lower Limits)

A common measure of the dispersity within a distribution is the standard deviation (σ), which defines the variation of points away from the average value.^49−51^ In this study, the deviation is determined based on an empirical probability rule that is applicable to any distribution type.^49^ Under this rule, at least 75% of the distribution values fall within two standard deviations (±2σ) away from the mean.^49^ The upper and lower molecular weight limits that correspond to a 75% confidence interval were determined from the cumulative molecular weight distribution plot of each peak.^25^

### 4.4 SANS Analysis

SANS analysis was carried out on a LARMOR small angle diffractometer at the ISIS Pulsed Neutron Source (STFC Rutherford Appleton Laboratory, Didcot, UK). Two mg mL^–1^ solutions of each conjugate were prepared by dissolving the conjugates in 5% d-DMSO and thereafter 95% D_2_O. The solutions were analyzed within 2 mm quartz cuvettes and their scattering cross section was measured over a *Q*-range of 0.004–0.5 Å^–1^, where *Q* is the scattering variable defined by the incident neutron wavelength (λ, 0.9–13.3 Å) and the scattering angle (θ).
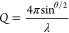
4

The conjugates’ spectra were collected at a sample–detector distance of 4.1 m, using a 6 mm × 8 mm beam size and 664 mm × 664 mm detector, width, and height, respectively. The resulting raw scattering data set was corrected for the detector efficiencies, sample transmission, and background scattering, then converted to scattering cross-section data (∂Σ/∂Ω vs *Q*) using the instrument-specific software. Thereafter, the data were translated to an absolute scale (cm^–1^) by using the scattering from a standard sample (a solid blend of hydrogenous and perdeuterated polystyrene). Data fitting was then performed using SASfit software, and in all cases, several fixed parameters were used. These include the sample concentration, the radius of the cyclic peptide core (3.75 Å),^80^ the background (0.002 cm^–1^), and the scattering length density (SLD) values for the solvent (6.33 × 10^–6^ Å^–2^), peptide core (1.42 × 10^–6^ Å^–2^) and polymer shell (Table S4). The SLD values were calculated using the NIST database calculator and based on the respective chemical structures. See Supporting Information, Section B.

## Supporting Information Available

Supplementary AF_4_ fractograms and calculations; SANS fitting information and plots; and sample synthesis characterization (PDF)
