# Supplementary material for: Investigating the Impact of Hydrophobic Polymer Segments on the Self-Assembly Behavior of Supramolecular Cyclic Peptide Systems via Asymmetric-Flow Field Flow Fractionation
Source: Macromolecules. 2023 Aug 26;56(17):6618–32. doi: 10.1021/acs.macromol.3c00442 (PMC10501196; doi:10.1021/acs.macromol.3c00442)
Supplement: Supplementary file 1 — ma3c00442_si_001.pdf [file ma3c00442_si_001.pdf]

Supplementary Information

Investigating the Impact of Hydrophobic Segments on the Self-Assembly Behaviour of Supramolecular Cyclic Peptide Systems via Asymmetric-Flow Field Flow Fractionation

Maria Kariuki, [a] Julia Y. Rho, [a] Stephen C. L. Hall, [b] and Sébastien Perrier.* [a, c, d]

a. Department of Chemistry, University of Warwick, Coventry CV4 7AL, UK

b. ISIS Neutron and Muon Source, Rutherford Appleton Laboratory, Didcot OX11 0QX

c. Warwick Medical School, University of Warwick, Coventry CV4 7AL, UK

d. Faculty of Pharmacy and Pharmaceutical Sciences, Monash University, Parkville, VIC 3052, Australia

* Corresponding author: s.perrier@warwick.ac.uk

A. AF$_4$

Additional AF$_4$-MALS-RI Fractograms of Conjugates

Figures S1 (a-d) below are the conjugate fractograms not included in the main text. The blue and red traces respectively represent signals from the refractive index (RI) and multi-angle light scattering (MALS) detectors. The molecular weight values (right y-axis) for each elution fraction are overlaid as black triangles.

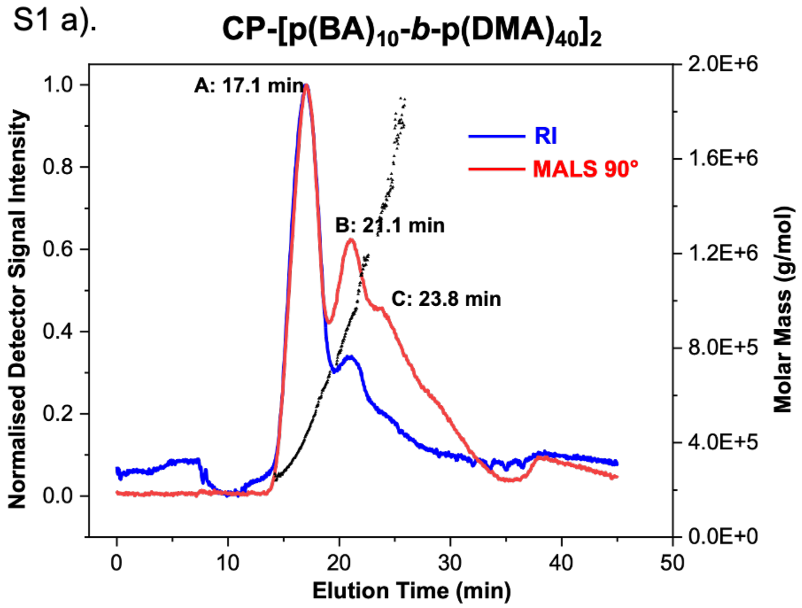

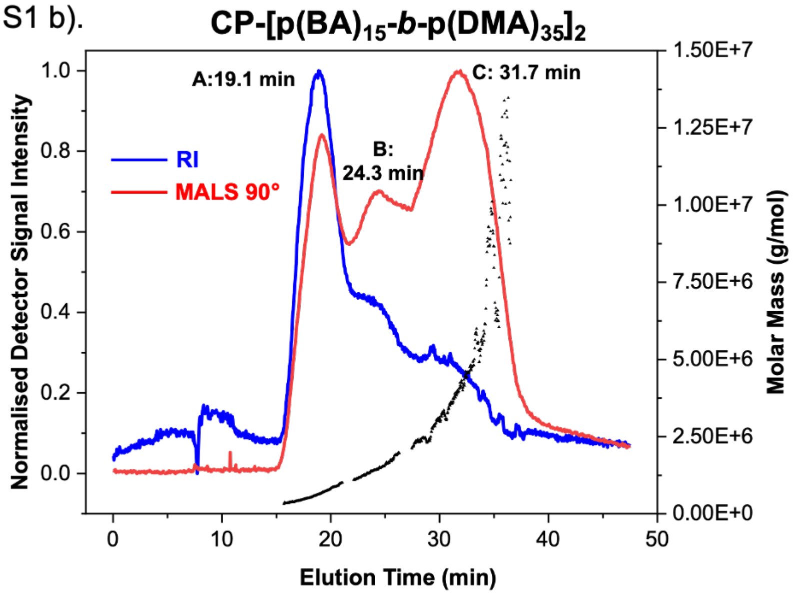

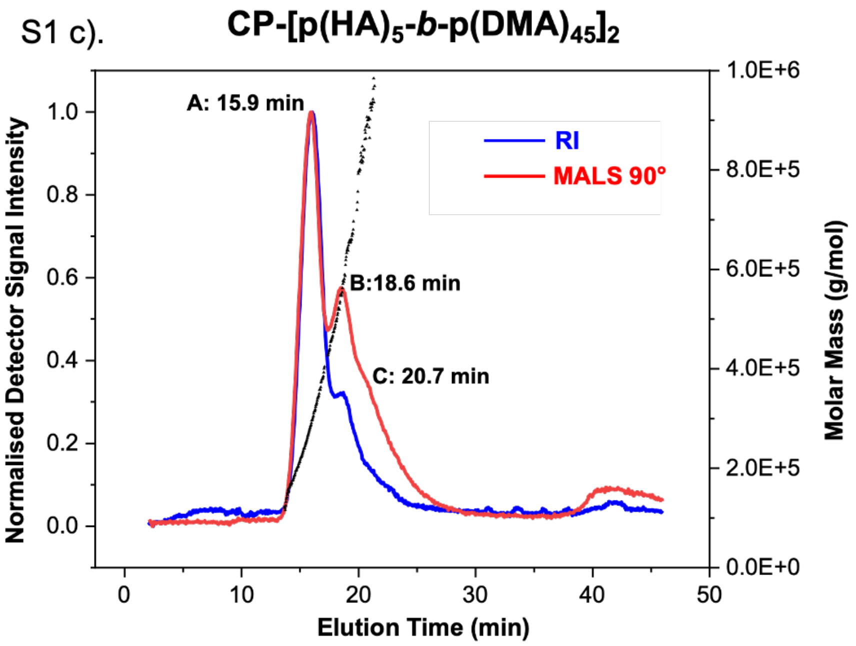

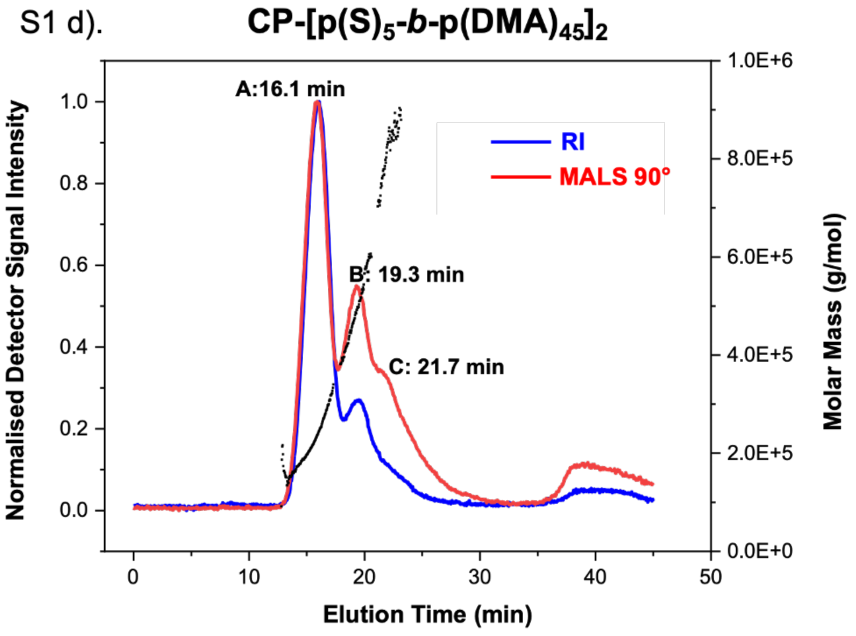

Figure S1. (a-d) AF_4-MALS-RI fractograms of conjugates: (**3**, CP-[p(BA)$_{10}$-*b*-p(DMA)$_{40}$]$_2$); (**4**, CP-[p(BA)$_{15}$-*b*-p(DMA)$_{35}$]$_2$); (**5**, CP-[p(HA)$_5$-*b*-p(DMA)$_{45}$]$_2$); (**8**, CP-[p(S)$_5$-*b*-p(DMA)$_{45}$]$_2$) respectively.

Polymer-Conjugate Comparisons

Figures S2 shows the comparisons of the amphiphilic conjugates to their control hydrophilic polymers as confirmation of the absence of both one-arm and two-arm unimers. The presence of structures due to the self-assembly of the unconjugated amphiphilic diblock copolymers is also ruled out. This is verified by the differences in the peak elution times and distribution profiles**.** It is also worth noting that the polymer standards and CP-polymer systems are distinguishable by UV-Vis detection at maxima

absorption wavelengths of 220, 280 and 309 nm. The polymers absorb light at 309 nm due to their trithiocarbonate group, while the conjugates absorb at all three wavelengths due to the combination of CP and polymer sensitivity. In all cases, measurements using a UV detector at 280 nm indicated similar distribution profiles as those measured by the RI detector therefore confirming that the populations were conjugate related.

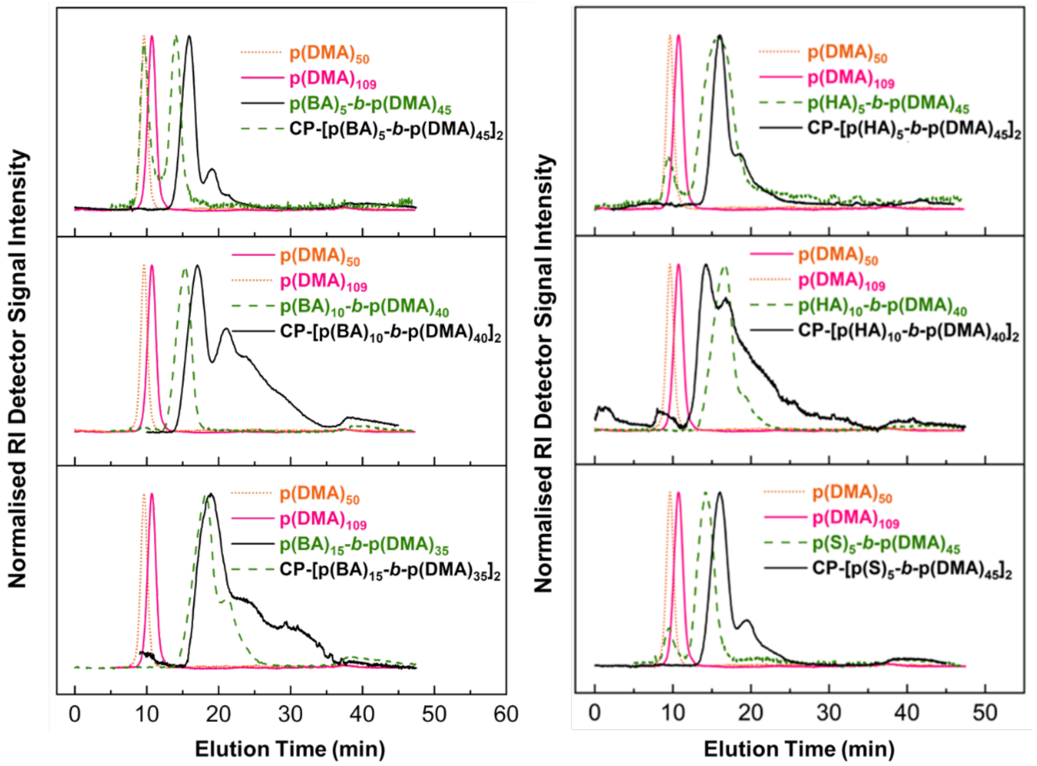

Figure S2. Comparison of the studied conjugates to their control polymers via their measured RI detector signals.

Conjugate Comparisons

Figures S3 highlights the fractogram comparisons of the amphiphilic conjugates by their hydrophobic monomers at 20 mol. % hydrophobicity. The results helped assign some of the peaks from the less soluble hexyl acrylate conjugate since quantitative analysis of this conjugate could not be performed.

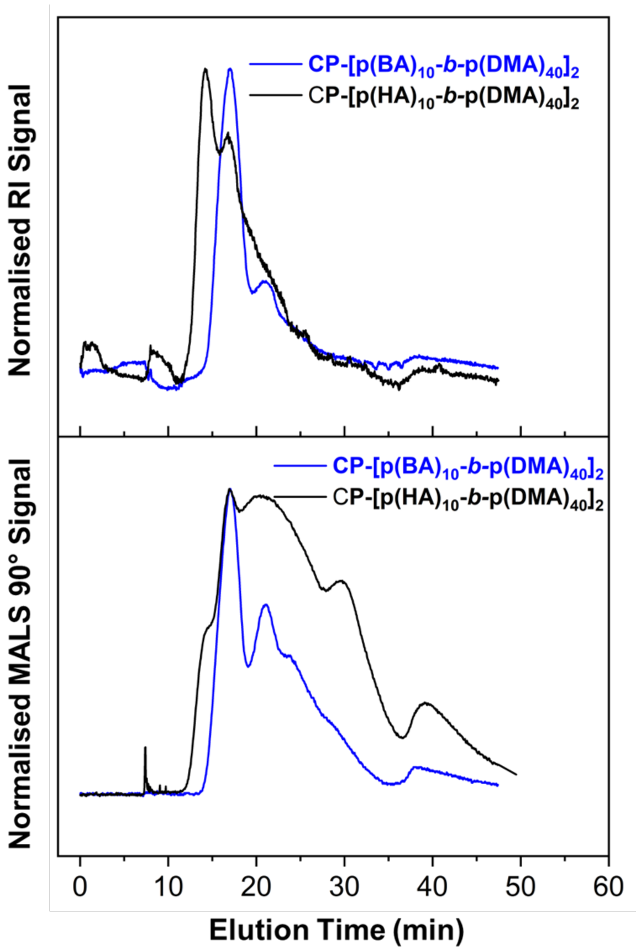

Figure S3. Fractogram comparison of the butyl acrylate (blue) and hexyl acrylate (black) conjugates at 20 mol.% hydrophobicity.

Solubility Images

Figures S4.4 (a-d) are the images of the conjugate solutions where precipitation was observed.

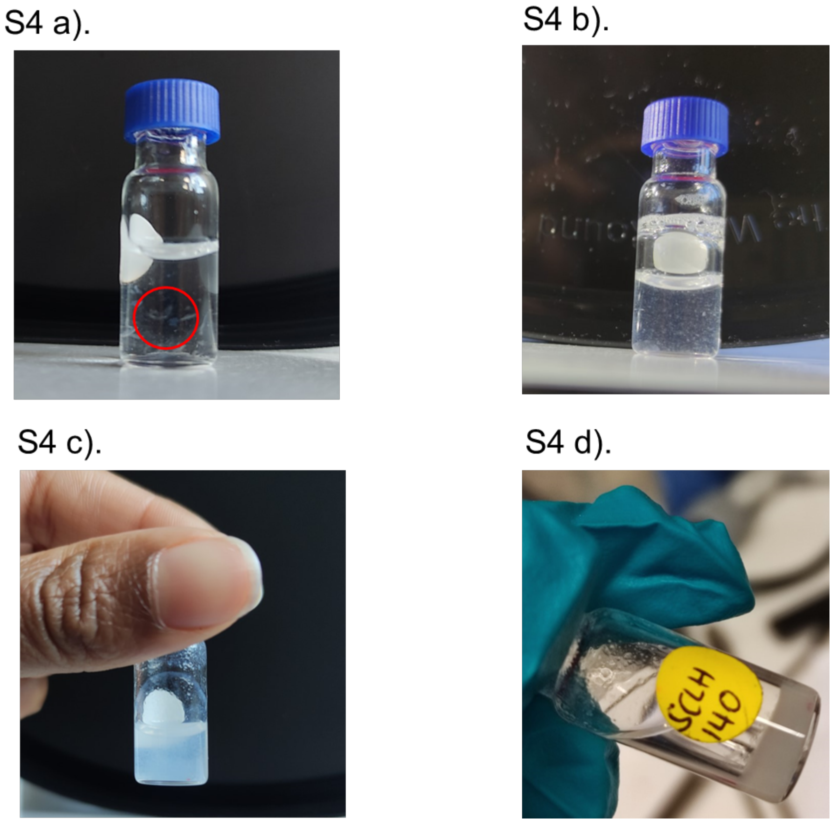

Figure S4. (a) Solution of conjugate (**4**, CP-[p(BA)$_{15}$-*b*-p(DMA)$_{35}$]$_2$) where gel-like aggregates are seen. (b) Colloidal solution of conjugate (**6**, CP-[p(HA)$_{10}$-*b*-p(DMA)$_{40}$]$_2$), following preparation. (c-d) Example images of the insoluble conjugates which heavily precipitated out of solution following preparation and sedimented after a few hours. These are: conjugates (**7**, CP-[p(HA)$_{15}$-*b*-p(DMA)$_{35}$]$_2$), (**9**, CP-[p(S)$_{10}$-*b*-p(DMA)$_{40}$]$_2$) and (**10**, CP-[p(BA)$_{15}$-*b*-p(DMA)$_{35}$]$_2$).

Method Reproducibility

Figure **S5** details example fractograms confirming good method reproducibility hence validating the collected AF_4 results and the system performance.

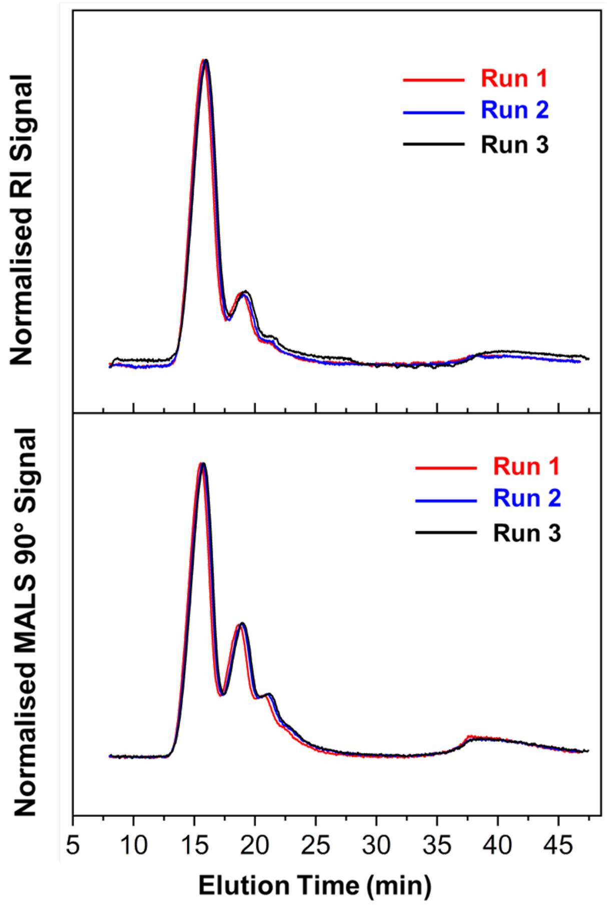

Figure S5. CP-[p(BA)$_5$-*b*-p(DMA)$_{45}$]$_2$ repeats as an example of good method reproducibility. Repeats of the other conjugates also showed consistency in the resulting fractogram profiles.

AF_4 Assessment of Self-Assembly Behaviour in Salt Containing & Salt-Free Solution

Figure S6 highlights a preliminary assessment of the H-bonding mediated self-assembly behaviour of example amphiphilic cyclic peptide-polymer systems in aqueous solutions containing physiological (Na+: 0.103 M and Cl-: 0.142 M) or zero concentrations of sodium chloride ions.

Indistinctive differences are observed from the distribution profiles. Precisely, similar peak populations were detected and supplementary calculations (**Table S1**) further indicated that the structures within the populations are equivalent in size and relative concentrations. Moreover, only slight shifts in the retention times are observed with

some conjugates and this can be related to the statistical nature of the AF_4 separation process due to inherent fluctuations in the environmental or instrumental conditions.

These results are consistent with a recent study by Paul and co-workers who employed molecular dynamics experiments to investigate the influence of different NaCl concentrations on the association of cyclic peptides in water.[1] Their study suggested that at low NaCl concentrations (≤ 0.68-3.42m, ~ 0.65-2.85 M), the Na^+, Cl^- ions impact association by screening electrostatic interactions between charged sites on the peptide backbone. Therefore, as our systems' constituent amino acids and polymers do not contain readily ionisable functional groups at the neutral pH of the aqueous NaCl (0.1 M) salt solution, it is logical that the salt does not contribute to the intermolecular association between cyclic peptide units.[2, 3] Furthermore, as determined by Bogunia *et al.*, any association behaviour driven by hydrophobic interactions should not be significantly influenced at physiological salt concentrations as the strength of the interactions is monotonic.[4, 5]

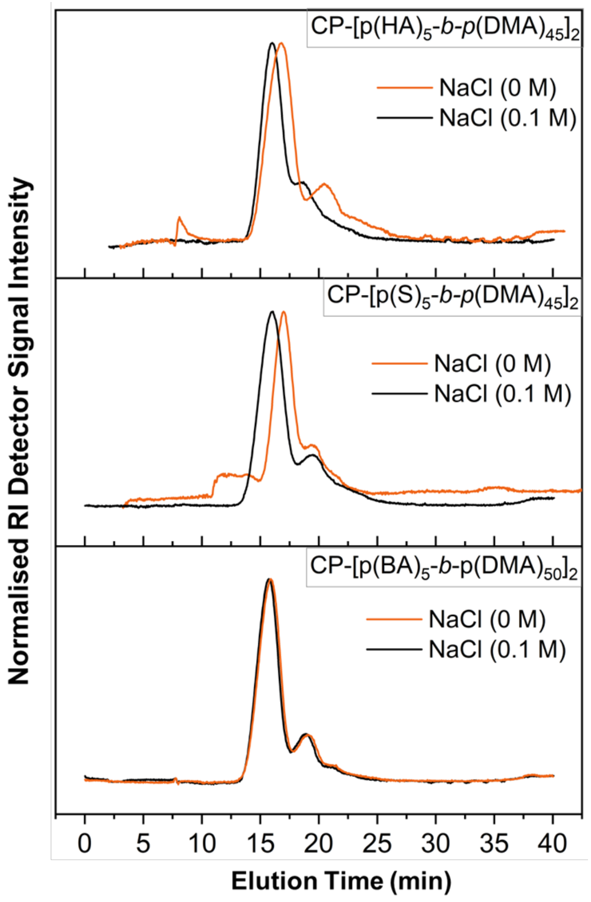

Figure S6. AF_4-RI traces of example conjugates measured in the aqueous measurement solvent (HPLC grade water) containing 0.1 and 0 M NaCl.

Table S1: Summary of the size parameters: molecular weight averages (M_w, M_n) and aggregation number (N_{agg}), of the detected nanotube populations featured in **Figure S6**. The tabulated values are the means of three data sets/repeats and have low relative standard errors (<5%).

NaCl Conc	Conjugate	†Population	% Relative Conc	*M_w	*M_n	‡$N_{agg, average}$
0.1 M	CP-[p(S)5-*b*-p(DMA)45]2	16.1 min	75.98	2.246 x 10^5	2.165 x 10^5	20
		19.3 min	18.26	4.719 x 10^5	4.650 x 10^5	41
		21.7 min	5.76	7.742 x 10^5	7.701 x 10^5	67
	CP-[p(HA)5-*b*-p(DMA)45]2	15.9 min	74.58	2.610 x 10^5	2.445 x 10^5	22
		18.6 min	20.67	5.317 x 10^5	5.220 x 10^5	44
		20.7 min	5.97	8.081 x 10^5	8.047 x 10^5	67
	CP-[p(BA)5-*b*-p(DMA)45]2	15.5 min	78.84	2.166 x 10^5	2.131 x 10^5	18
		18.7 min	16.48	4.327 x 10^5	4.279 x 10^5	37
		20.7 min	4.67	6.198 x 10^5	6.150 x 10^5	53
0 M	CP-[p(S)5-*b*-p(DMA)45]2	16.9 min	76.2	2.228 x 10^5	2.142 x 10^5	19
		19.5 min	17.9	4.653 x 10^5	4.592 x 10^5	41
		¤-	-	-	-	-
	CP-[p(HA)5-*b*-p(DMA)45]2	16.8 min	75.51	2.731 x 10^5	2.585 x 10^5	23
		20.4 min	19.48	5.207 x 10^5	5.124 x 10^5	41
		23.5 min	5.01	8.091 x 10^5	8.069 x 10^5	67
	CP-[p(BA)5-*b*-p(DMA)45]2	15.8 min	79.56	2.187 x 10^5	2.153 x 10^5	19
		18.9 min	16.34	4.435 x 10^5	4.392 x 10^5	38
		21.0 min	4.10	6.698 x 10^5	6.690 x 10^5	57

†Population peaking at the specified elution time. *Common units: Da or g mol^{-1}; ¤ The detected MALS signals have a low S/N ratio hence calculated weight values omitted due to lower accuracy. ‡ Calculated from the weight average molecular weight (M_w); Formula [$N_{agg} = M_{w(assembly)} \div MW_{(unimer)}$].

∂n/∂c Calculations

Due to the much lower cyclic peptide yield attainable during synthesis (mg), in addition to the higher synthesis costs, the unconjugated polymers were used to estimate the ∂n/∂c of the conjugates based on three general assumptions. The first assumption is that the ∂n/∂c values of the polymers are representative of the conjugates because the polymers make up the majority of the conjugate structure. It was also assumed, in line with theory, that the ∂n/∂c would vary negligibly with increasing molecular weight (from ≥ 10^3 Da) hence the obtained values would be valid for both unimers and nanotube assemblies.[6-8] Lastly, with regards to the amphiphilic diblock copolymers, it was hypothesised that the contributions of each block were proportionately accounted for in the obtained final values.[6, 7] This assumption was necessary as the ∂n/∂c of the hydrophobic blocks could not be individually determined in the aqueous solution used for analysis. It is also worth noting that an online approach was implemented as it is fast, straightforward and requires the preparation of less sample in comparison to batch analyses.[9, 10] In addition ,the group has validated the accuracy of this approach using common polymer standards

whose ∂n/∂c values are available in literature. The mean results obtained from at least 3 consistent measurements are shown in **Table S2**.

Table S2: ∂n/∂c values of the conjugates at 25°C in NaCl (0.1M) solution. The RI laser wavelength is within the Infrared-Ultraviolet range.

Conjugate	Determined Using	Mean ∂n/∂c (mL/g)	SD, σ(mL/g)
CP-[p(DMA)$_{50}$]$_2$	pDMA$_{50}$	0.2105	±0.0027
CP-[p(BA)$_5$-*b*-p(DMA)$_{45}$]$_2$	p(BA)$_5$-*b*-p(DMA)$_{45}$	0.1714	±0.0017
CP-[p(BA)$_{10}$-*b*-p(DMA)$_{40}$]$_2$	p(BA)$_{10}$-*b*-p(DMA)$_{40}$	0.1696	±0.0018
CP-[p(BA)$_{15}$-*b*-p(DMA)$_{35}$]$_2$	p(BA)$_{15}$-*b*-p(DMA)$_{35}$	0.1715	±0.0011
CP-[p(HA)$_5$-*b*-p(DMA)$_{45}$]$_2$	p(HA)$_5$-*b*-p(DMA)$_{45}$	0.1854	±0.0027
CP-[p(S)$_5$-*b*-p(DMA)$_{45}$]$_2$	p(S)$_5$-*b*-p(DMA)$_{45}$	0.1872	±0.0013

Standard Error (SE) Calculations

The standard error (SE) is a measure of the reliability of the population mean and can be expressed as a percentage, referred to as the relative standard error (RSE). A small SE/RSE (≤ 25%) is an indication that the population mean is an accurate reflection of the samples within a population.[11]

Table S3: Relative SE calculations of the molecular weight means from three sample repeats.

Conjugate	§ Population	$^{*†}M_w$	*RSE*	$^{*†}M_n$	*RSE*
CP-[p(DMA)$_{50}$]$_2$	9.8 min	1.383 x 10^4	1.04%	1.238 x 10^4	0.84%
	A: 16.2 min	2.446 x 10^5	3.22%	2.066 x 10^5	2.48%
CP-[p(BA)$_5$-*b*-p(DMA)$_{45}$]$_2$	A: 15.5 min	2.166 x 10^5	0.48%	2.131 x 10^5	0.53%
	B: 18.7 min	4.327 x 10^5	2.36%	4.279 x 10^5	2.44%
	C: 20.7 min	6.198 x 10^5	5.28%	6.150 x 10^5	5.35%
CP-[p(BA)$_{10}$-*b*-p(DMA)$_{40}$]$_2$	A: 17.1 min	4.182 x 10^5	0.77%	3.933 x 10^5	0.95%
	B: 21.1 min	9.494 x 10^5	0.72%	9.325 x 10^5	0.76%
	C: 23.8 min	1.638 x 10^6	3.95%	1.617 x 10^6	3.82%
CP-[p(BA)$_{15}$-*b*-p(DMA)$_{35}$]$_2$	A: 19.1 min	6.110 x 10^5	0.48%	5.638 x 10^5	0.61%
	B: 23.4 min	1.422 x 10^6	1.27%	1.387 x 10^6	1.19%
	C: 31.7 min	3.626 x 10^6	6.37%	3.208 x 10^6	3.30%
CP-[p(HA)$_5$-*b*-p(DMA)$_{45}$]$_2$	A: 15.9 min	2.610 x 10^5	4.50%	2.445 x 10^5	5.37%
	B: 18.6 min	5.317 x 10^5	4.33%	5.220 x 10^5	4.42%
	C: 20.7 min	8.081 x 10^5	11.38%	8.047 x 10^5	11.24%
CP-[p(S)$_5$-*b*-p(DMA)$_{45}$]$_2$	A: 16.1 min	2.246 x 10^5	0.41%	2.165 x 10^5	0.58%
	B: 19.3 min	4.719 x 10^5	1.07%	4.650 x 10^5	0.90%
	C: 21.7 min	7.742 x 10^5	2.14%	7.701 x 10^5	2.13%

§ Populations eluting at the specified time, alphabetised populations refer to the nanotube assemblies. *Common units: Da or gmol^{-1}. † Represents the mean of the values from three sample repeats (population mean). RSE = (SE ÷ $\bar{x}_p$) and SE = (σ_p ÷ √N); where σ_p is the standard deviation of the samples within the population, N is the population size and $\bar{x}p$ is the population mean.

B. SANS

The morphology of the conjugates were evaluated using SANS and **Figures S7 (a-c)** show reduced scattering data and fits. The insoluble conjugates were not characterised as their propensity to sediment would result in inconclusive data.

With the exception of conjugate (**1**, CP-[p(DMA)$_{50}$]$_2$), all featured conjugates were fit to a hairy core-shell cylinder model using SAS view software. The model description is as follows:[12]

$$P(q) = N^2\beta_s^2 F_{s(q)} + N^2\beta_c^2 F_{c(q)} + 2N^2\beta_s\beta_c S_{sc(q)} + N(N-1)\beta_c^2 S_{cc(q)}$$

$$F_{s(q,R,L)} = F_{cs(q,R)} F_{L(q,L)}$$

$$F_{cs(q,R)} = \left(\frac{2B_1 qR}{qR}\right)^2 ; \qquad F_{L(q,L)} = \left(\frac{2Si(qL)}{qL}\right) - \frac{4sin^2(\frac{qL}{2})}{q^2L^2}$$

$$F_{c(q)} = \frac{2[\exp(q^2R_g^2) - 1 + q^2R_g^2}{q^4R_g^4} \qquad Si(x) = \int_0^x \frac{sint}{t} dt$$

$$S_{cc(q)} = \psi(qR_g)^2 B_0[q(R + dR_g)]^2 F_{L(q,L)}$$

$$S_{sc(q)} = \psi(qR_g)\frac{2B_1 qR}{qR} B_0[q(R + dR_g)] F_{L(q,L)}$$

$$\psi(qR_g) = \frac{1 - exp(-qR_g)}{qR_g}$$

Where N is the aggregation number; $\beta_s = V_s\,(\rho_s - \rho_{solv})$ and $\beta_c = V_c\,(\rho_c - \rho_{solv})$ are the total excess scattering lengths of a block in the cylindrical core and in the corona, respectively; V_s and V_c are the volumes of a block in the core and in the corona, respectively; ρ_s and ρ_c are the corresponding scattering length densities and ρ_{solv} is the scattering length density of the surrounding solvent; B_1 and B_0 are the first and zero order Bessel function respectively and R_g is the gyration radius of the block of the corona.

A combined model including a core-shell cylinder and mono-Gaussian coil form factor was used to fit conjugate (**1**, CP-[p(DMA)$_{50}$]$_2$). The mono-Gaussian model is defined as follows:[13]

$$P(q) = \frac{2[\exp(-Z) + Z - 1]}{Z^2} \quad , Z = (qR_g)^2$$

S7 a).

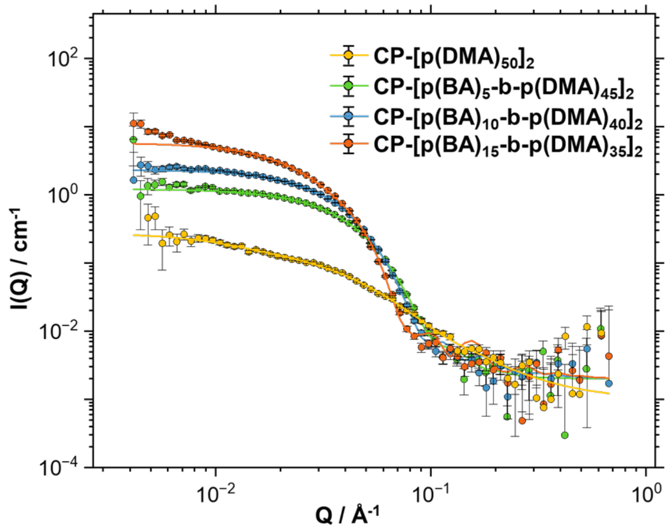

CP-[p(DMA)50]2
CP-[p(BA)5-b-p(DMA)45]2
CP-[p(BA)10-b-p(DMA)40]2
CP-[p(BA)15-b-p(DMA)35]2
I(Q) / cm-1
Q / Å-1

S7 b).

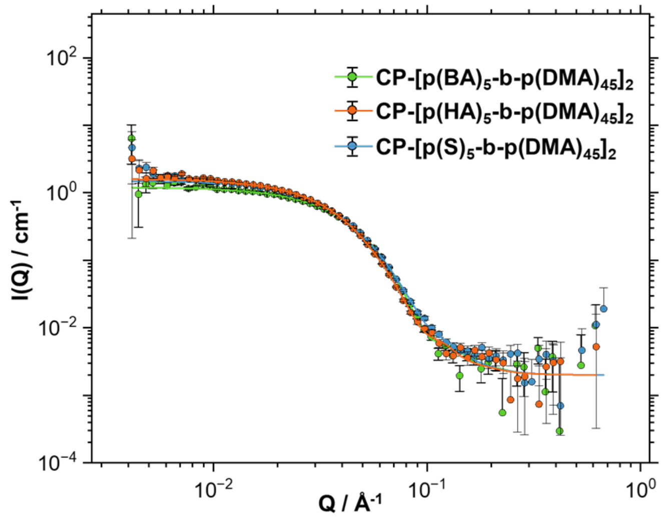

CP-[p(BA)5-b-p(DMA)45]2
CP-[p(HA)5-b-p(DMA)45]2
CP-[p(S)5-b-p(DMA)45]2
I(Q) / cm-1
Q / Å-1

S7 c).

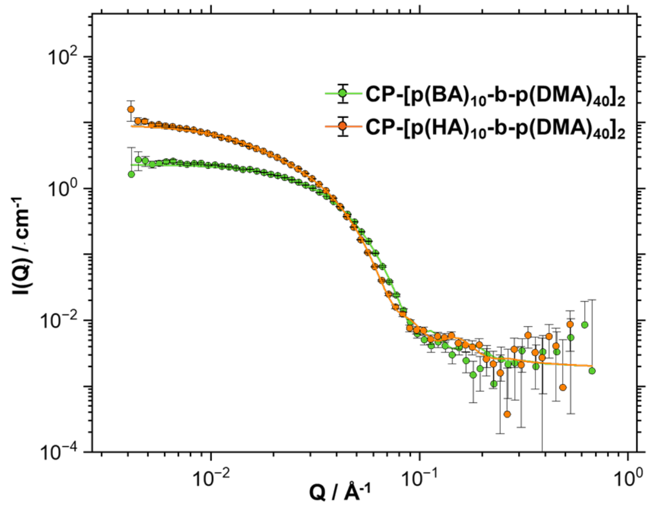

CP-[p(BA)10-b-p(DMA)40]2
CP-[p(HA)10-b-p(DMA)40]2
I(Q) / cm-1
Q / Å-1

Figure S7: Comparison of the SANS scattering profiles of the conjugates in 95% D_2O and 5% d-DMSO. CP-[p(DMA)$_{50}$]$_2$ was fit using a combined model including a core-shell cylinder and Gaussian coil form factor. The remaining conjugates were fit to a core-shell cylinder model. The fits were considered statistically reliable as their Chi^2 values were <20.

Table S4: Relevant SANS fitting and calculated parameters of the conjugates

Conjugate	SLD, $_{polymer}$ (x 10^{-6} A^{-2})	Length $_{(cylinder)}$ (Å)
CP-[p(DMA)$_{50}$]$_2$	0.80	38.65 ± 10.45
CP-[p(BA)$_5$-*b*-p(DMA)$_{45}$]$_2$	5.89	41.68 ± 2.04
CP-[p(BA)$_{10}$-*b*-p(DMA)$_{40}$]$_2$	5.98	52.43 ± 1.66
CP-[p(BA)$_{15}$-*b*-p(DMA)$_{35}$]$_2$	6.03	116.38 ± 1.46
CP-[p(HA)$_5$-*b*-p(DMA)$_{45}$]$_2$	4.75	55.88 ± 2.33
CP-[p(HA)$_{10}$-*b*-p(DMA)$_{40}$]$_2$	5.91	225.53 ± 3.30
CP-[p(S)$_5$-*b*-p(DMA)$_{45}$]$_2$	2.13	57.57 ± 2.33

C. Synthesis Characterisation

Peptide Characterisation Data

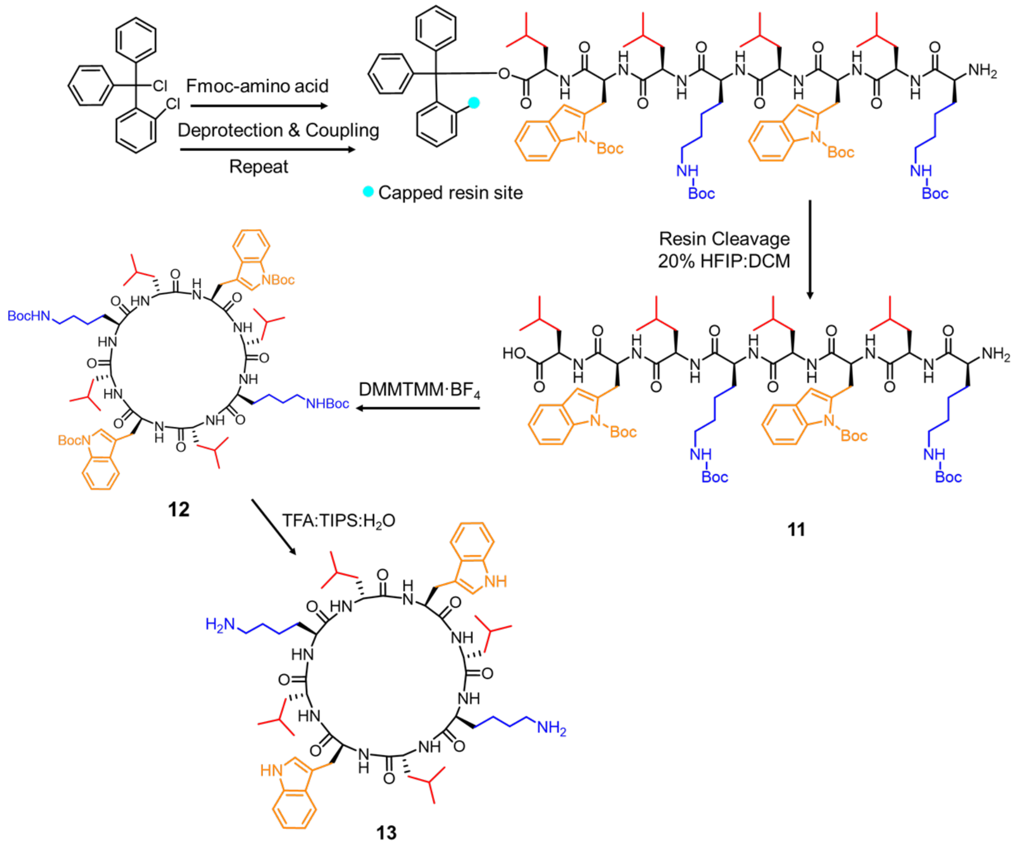

Scheme S1: Simplified scheme illustrating the synthesis of the linear octa-peptide (**11**) using solid phase peptide synthesis (SPPS), the cyclisation of the linear peptide into a cyclic peptide (**12**) and the Boc-deprotection of the cyclic peptide to allow post-modification (**13**).

Table S5: ESI-ToF-MS Characterisation of the Linear and Cyclic Peptide Products of amino acid sequence: (D-Leu-L-Trp- D-Leu-L-Lys-)2.

Label	Compound	ESI Peak ($gmol^{-1}$)		
		Attribution	Calculated	Found
**11**	Linear peptide	$[M+Na]^+$	1522.9	1522.8
		$[M+H]^+$	1500.9	1500.8
**12**	Boc-Protected cyclic peptide	$[M+Na]^+$	1504.89	1503. 8
**13**	Deprotected cyclic peptide	$[M+Na]^+$	1104.42	1104.5
		$[M+H]^+$	1082.42	1081.5

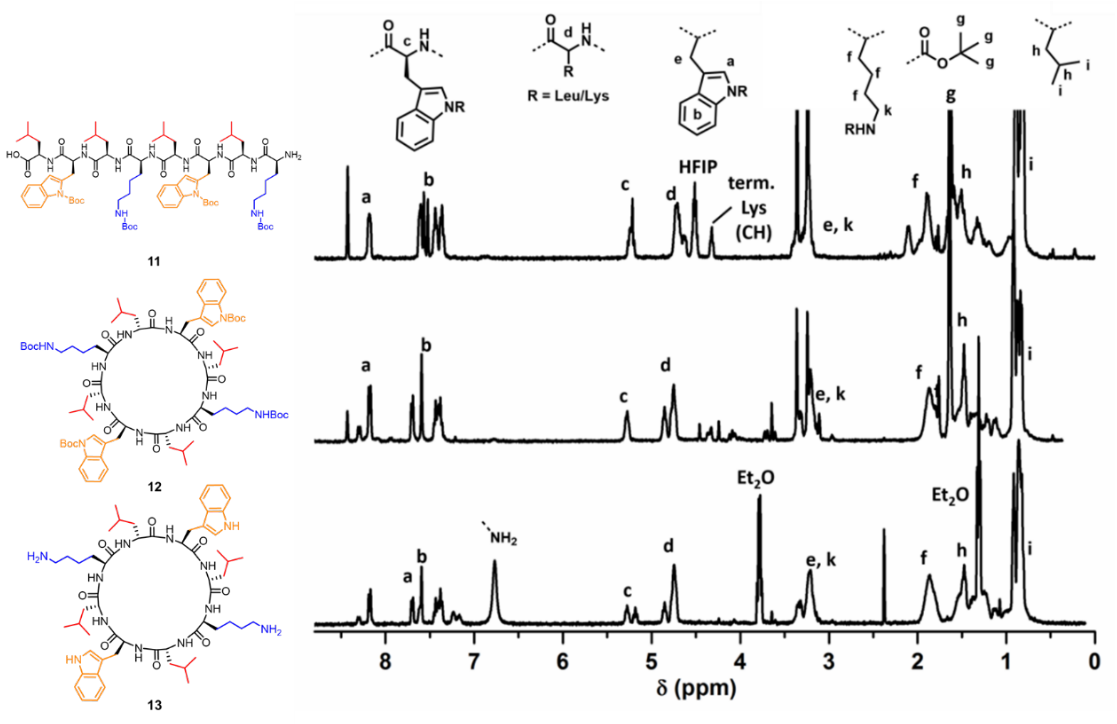

Figure S8: ^{1}H-NMR (*d*-TFA) spectra of the linear and cyclic peptide products with amino acid sequence: (D-Leu-L-Trp- D-Leu-L-Lys-)2. Adapted from Mansfield *et al.*[14]

Polymer Characterisation Data

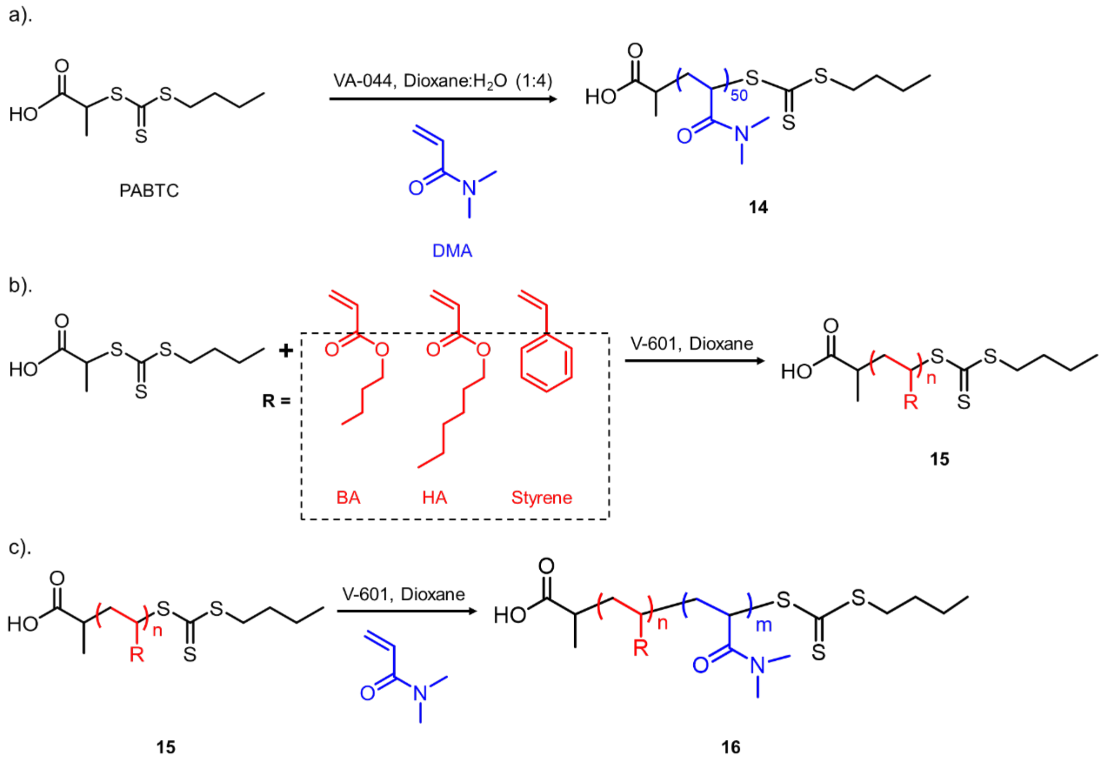

Scheme S2: (a) Synthesis of homopolymer pDMA with a chain length of 50 (**14**). (b-c) Preparation of diblock co-polymers beginning with the synthesis of the first hydrophobic block (**15**) then the chain extension of the second block (**16**).

Table S6: SEC characterisation data of the polymers

Polymer	^{a,b} *MW, theo* (10^3)	^{b} $M_{w, SEC}$ (10^3)	^{b} $M_{n, SEC}$ (10^3)	*Đ*
Hydrophilic Homopolymer				
†p(DMA)$_{50}$	4.96	4.55	3.93	1.16
Hydrophobic Blocks				
§p(BA)$_5$	0.88	1.28	0.84	1.54
§p(BA)$_{10}$	1.52	2.03	1.29	1.57
§p(BA)$_{15}$	2.16	2.84	1.82	1.56
†p(HA)$_5$	1.05	*0.32	*0.30	*1.09
†p(HA)$_{10}$	1.98	*0.31	*0.28	*1.10
†p(HA)$_{15}$	2.98	*0.29	*0.26	*1.12
§p(S)$_5$	0.79	0.93	0.84	1.11
§p(S)$_{10}$	1.35	1.45	1.27	1.14
§p(S)$_{15}$	1.85	1.94	1.71	1.14
Diblock Copolymers				
†p(BA)$_5$-*b*-p(DMA)$_{45}$	5.34	6.23	5.52	1.13
†p(BA)$_{10}$-*b*-p(DMA)$_{40}$	5.49	5.87	5.20	1.13
†p(BA)$_{15}$-*b*-p(DMA)$_{35}$	5.63	6.29	5.62	1.12
†p(HA)$_5$-*b*-p(DMA)$_{45}$	5.49	7.10	6.14	1.16
†p(HA)$_{10}$-*b*-(DMA)$_{40}$	6.12	5.61	4.87	1.15
†p(HA)$_{15}$-*b*-(DMA)$_{35}$	6.42	4.60	4.03	1.14
†p(S)$_5$-*b*-p(DMA)$_{45}$	5.30	7.32	6.39	1.15
†p(S)$_{10}$-*b*-p(DMA)$_{40}$	5.23	5.89	5.02	1.17
†p(S)$_{15}$-*b*-p(DMA)$_{35}$	5.63	5.56	4.83	1.15

^{a} Calculated based on DP (% Conversion x Target DP) determined from ^{1}H NMR. ^{b} Common units = Da or gmol^{-1}. † Eluent = DMF (+ 5mM NH_4BF_4), calibration standards = PMMA standards. § Eluent = THF (+ 0.01 % BHT), calibration standards = PMMA and PS standards. * Inconclusive as some of the sample's peak fractions overlapped with the system peak.

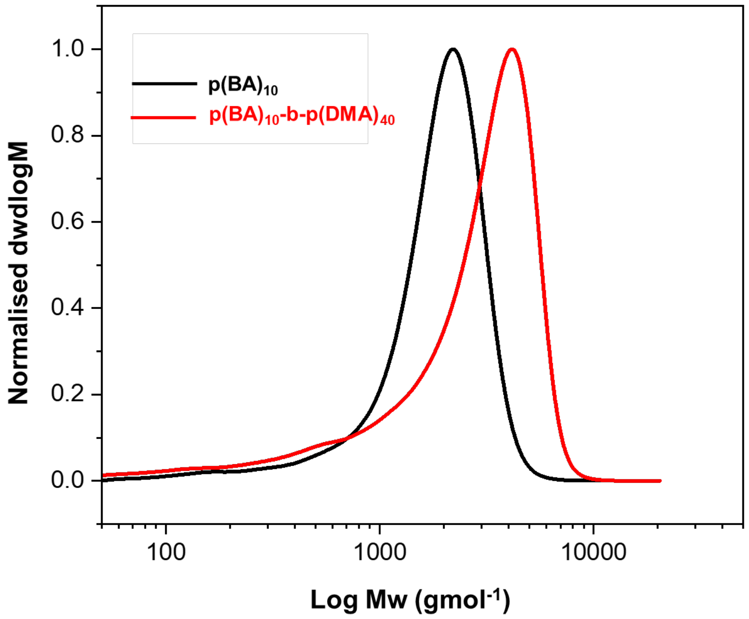

Figure S9: Example SEC trace of a successful chain extension from homopolymer to diblock copolymer.

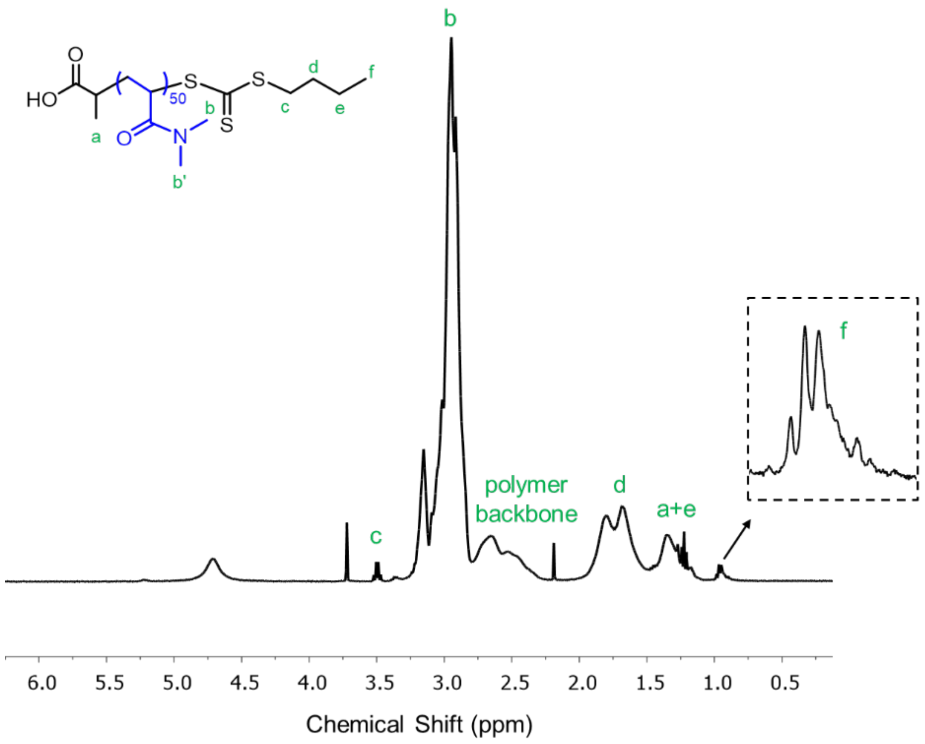

Figure S10: ^{1}H NMR ($CDCl_3$) spectrum of the pDMA homopolymer.

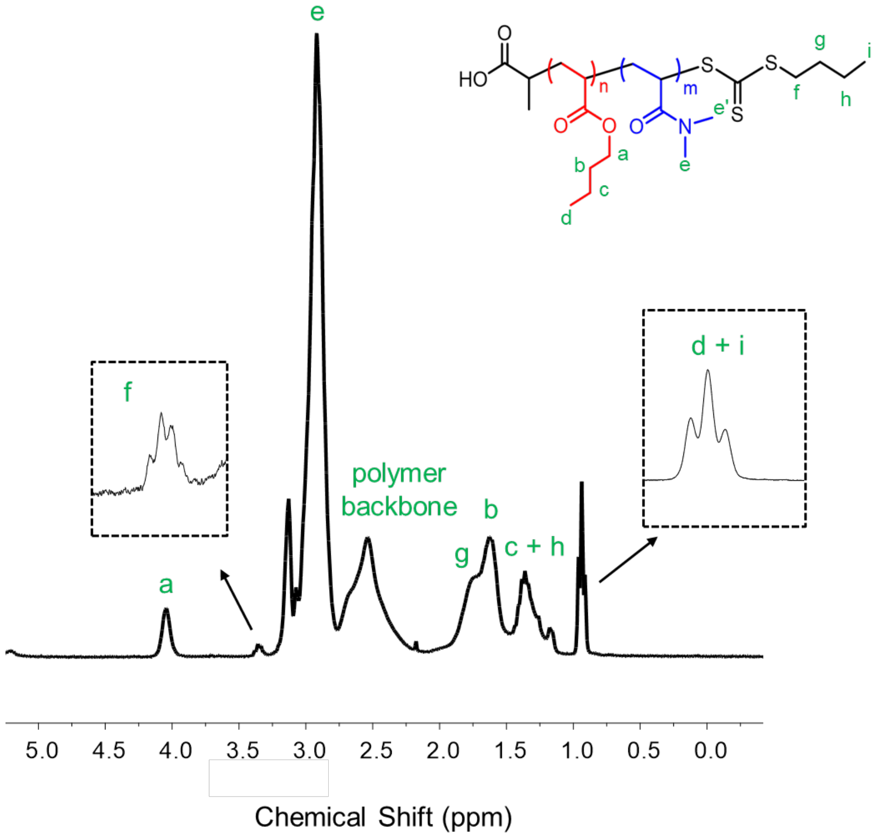

Figure S11: Example ^{1}H NMR ($CDCl_3$) spectrum of the pBA-*b*-pDMA diblock copolymers.

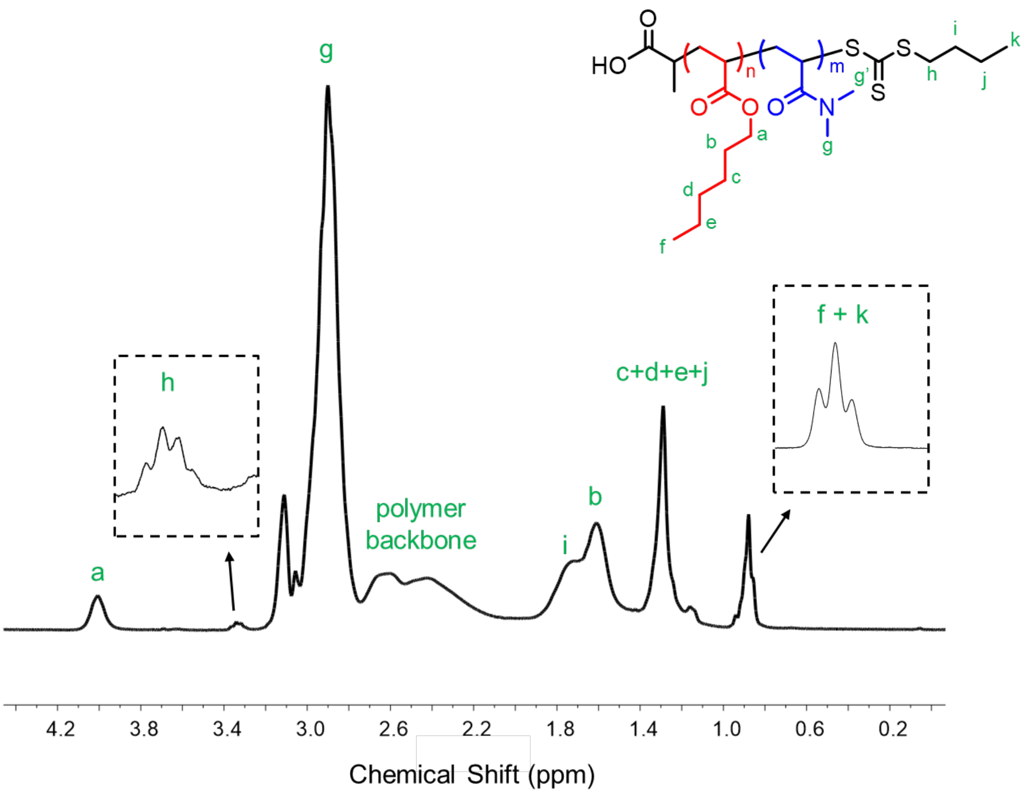

Figure S12: Example ^{1}H NMR ($CDCl_3$) spectrum of the pHA-*b*-pDMA diblock copolymers.

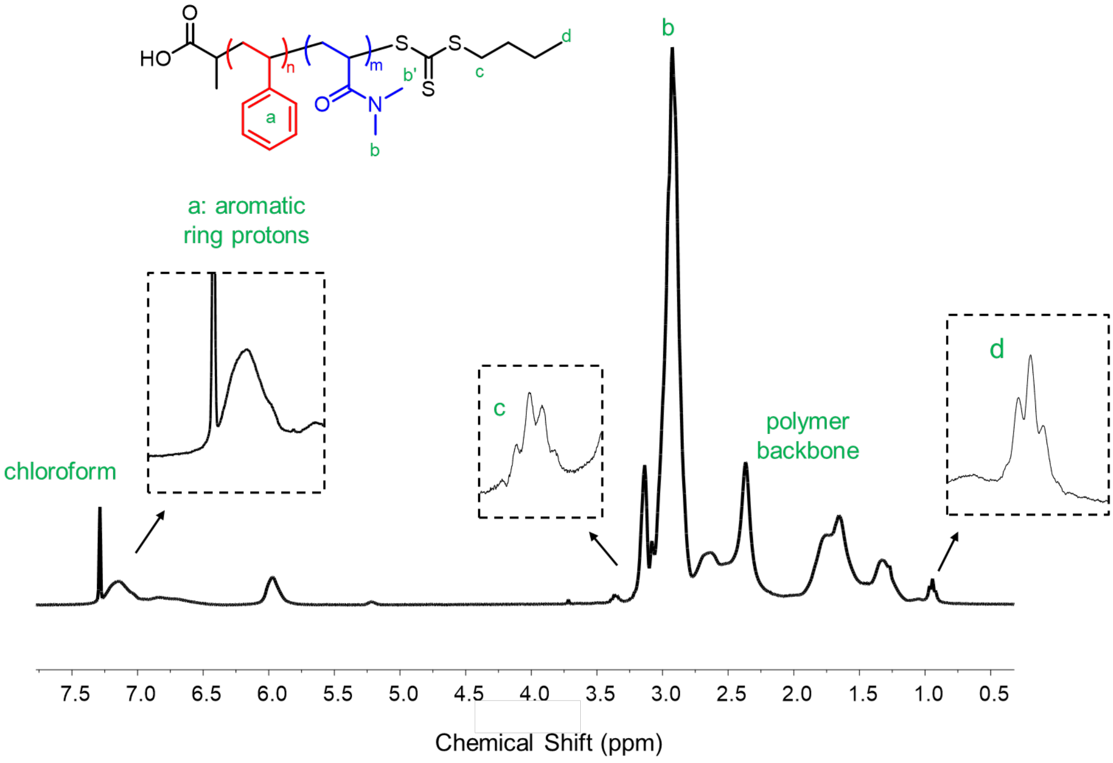

Figure S13: Example ^{1}H NMR ($CDCl_3$) spectrum of the pS-*b*-pDMA diblock copolymers.

Conjugate Characterisation Data

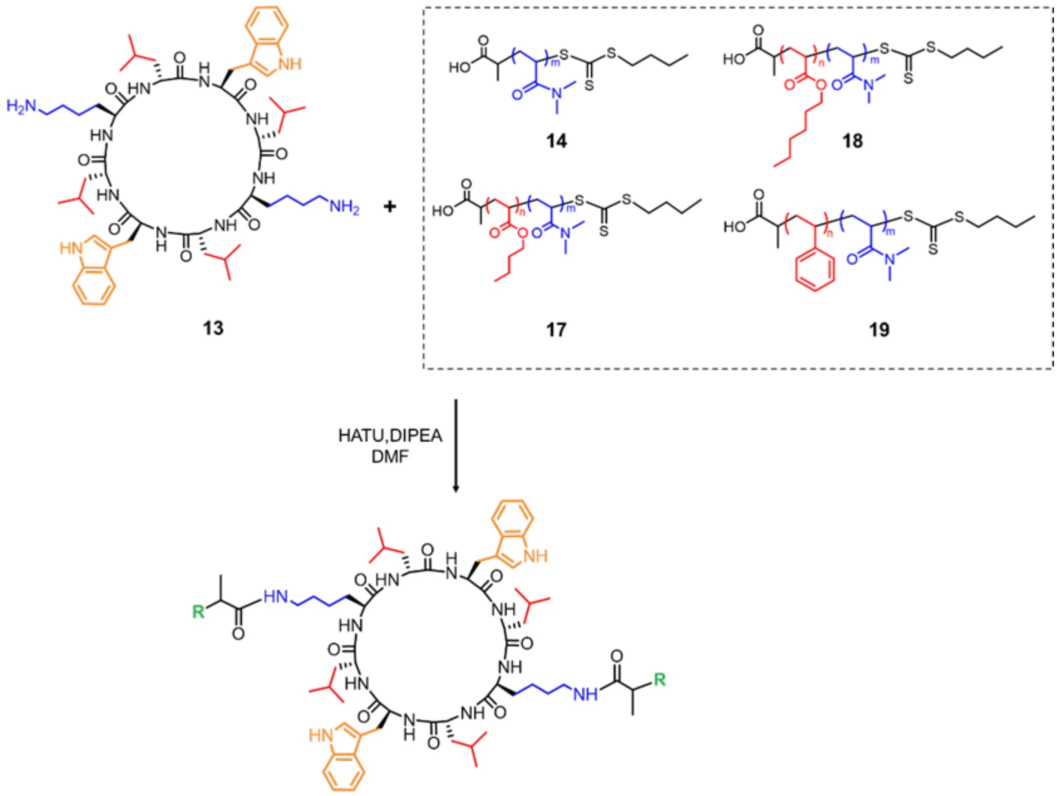

Scheme S3: Polymer conjugation to the cyclic peptide. R = rest of polymer structure.

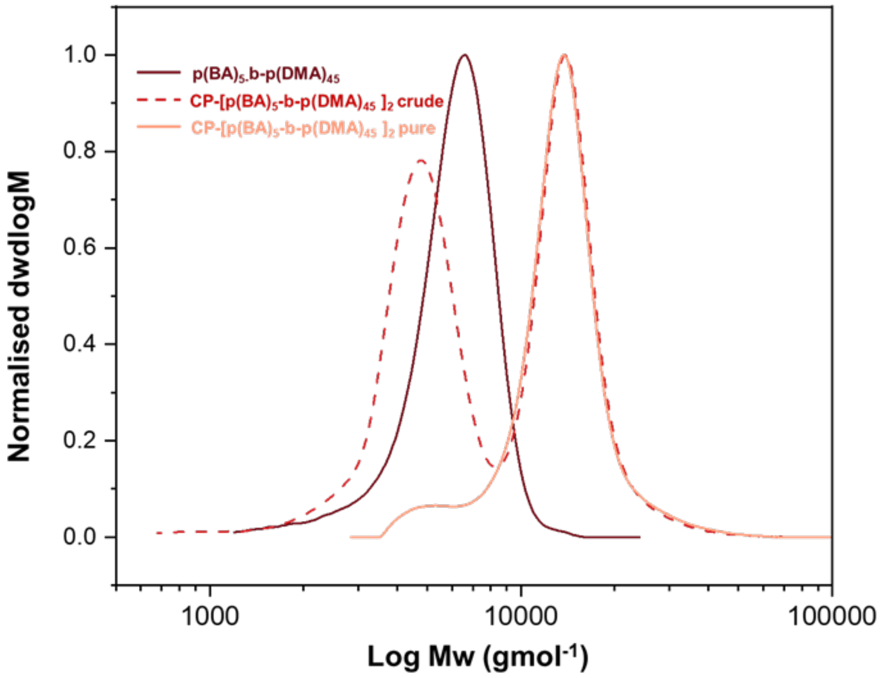

Figure S14: Example SEC trace (DMF) showing the successful conjugation of two polymer arms onto a cyclic peptide and subsequent purification.

Table S7: SEC characterisation of the conjugates used in this study.

Label	Conjugate	[a,b] $M_{w,theo}$ (10^4)	[b,c] $M_{w,SEC}$ (10^4)	[b,c] $M_{n,SEC}$ (10^4)	$Đ$
**1**	CP-[p(DMA)$_{40}$]$_2$	1.144	1.46	1.27	1.15
**2**	CP-[p(BA)$_5$-*b*-p(DMA)$_{45}$]$_2$	1.173	1.40	1.22	1.15
**3**	CP-[p(BA)$_{10}$-*b*-p(DMA)$_{40}$]$_2$	1.202	1.47	1.29	1.14
**4**	CP-[p(BA)$_{15}$-*b*-p(DMA)$_{35}$]$_2$	1.231	1.66	1.49	1.12
**5**	CP-[p(HA)$_5$-*b*-p(DMA)$_{45}$]$_2$	1.201	1.61	1.37	1.17
**6**	CP-[p(HA)$_{10}$-*b*-p(DMA)$_{40}$]$_2$	1.258	1.69	1.50	1.13
**7**	CP-[p(HA)$_{15}$-*b*-p(DMA)$_{35}$]$_2$	1.315	1.71	1.53	1.12
**8**	CP-[p(S)$_5$-*b*-p(DMA)$_{45}$]$_2$	1.149	1.76	1.45	1.22
**9**	CP-[p(S)$_{10}$-*b*-p(DMA)$_{40}$]$_2$	1.154	1.79	1.40	1.28
**10**	CP-[p(S)$_{15}$-*b*-p(DMA)$_{35}$]$_2$	1.159	1.79	1.41	1.27

[a] Estimated using ChemDraw software analysis. [b] Common units = Da or $gmol^{-1}$. [c] Determined by size exclusion chromatography (SEC) calibrated with PMMA standards and using dimethylformamide (DMF) as eluent with 0.1% LiBr additives.

References

1. Moral, R.; Paul, S., Influence of salt and temperature on the self-assembly of cyclic peptides in water: a molecular dynamics study. *Physical Chemistry Chemical Physics* **2023,** *25* (7), 5406-5422.

2. Kocak, G.; Tuncer, C.; Bütün, V., pH-Responsive polymers. *Polymer Chemistry* **2017,** *8* (1), 144-176.

3. Pace, C. N.; Grimsley Gr Fau - Scholtz, J. M.; Scholtz, J. M., Protein ionizable groups: pK values and their contribution to protein stability and solubility. *Journal of Biological Chemistry* **2009,** *284* (20), 13285-13289.

4. Bogunia, M.; Makowski, M., Influence of Ionic Strength on Hydrophobic Interactions in Water: Dependence on Solute Size and Shape. *The Journal of Physical Chemistry B* **2020,** *124* (46), 10326-10336.

5. Bogunia, M.; Liwo, A.; Czaplewski, C.; Makowska, J.; Giełdoń, A.; Makowski, M., Influence of Temperature and Salt Concentration on the Hydrophobic Interactions of Adamantane and Hexane. *The Journal of Physical Chemistry B* **2022,** *126* (3), 634-642.

6. Coto, B.; Escola, J. M.; Suárez, I.; Caballero, M. J., Determination of dn/dc values for ethylene–propylene copolymers. *Polymer Testing* **2007,** *26* (5), 568-575.

7. Wu, C. S., *Handbook Of Size Exclusion Chromatography And Related Techniques: Revised And Expanded*. 2nd ed.; Marcel Dekker: USA, 2003; Vol. 91.

8. Elias, H.-G., Scattering Methods. *Macromolecules* **2008**, 139-171.

9. Panalytical, M. Principles of Triple Detection GPC/SEC. https://www.malvernpanalytical.com/en/learn/knowledge-center/whitepapers/wp151119principlestripledetectiongpc (accessed 23/06/2022).

10. Podzimek, S., *Light Scattering, Size Exclusion Chromatography and Asymmetric Flow Field Flow Fractionation: Powerful Tools for the Characterization of Polymers, Proteins and Nanoparticles*. John Wiley & Sons: New Jersey, 2011.

11. Rumsey, D. J., *Statistics For Dummies*. 2nd ed.; Wiley: Indianna, 2016.

12. Yang, J.; Song, J.-I.; Song, Q.; Rho, J. Y.; Mansfield, E. D. H.; Hall, S. C. L.; Sambrook, M.; Huang, F.; Perrier, S., Hierarchical Self-Assembled Photo-Responsive Tubisomes from a Cyclic Peptide-Bridged Amphiphilic Block Copolymer. *Angewandte Chemie International Edition* **2020,** *59* (23), 8860-8863.

13. Debye, P., Molecular-weight Determination by Light Scattering. *The Journal of Physical and Colloid Chemistry* **1947,** *51* (1), 18-32.

14. Mansfield, E. D. H.; Hartlieb, M.; Catrouillet, S.; Rho, J. Y.; Larnaudie, S. C.; Rogers, S. E.; Sanchis, J.; Brendel, J. C.; Perrier, S., Systematic study of the structural parameters affecting the self-assembly of cyclic peptide–poly(ethylene glycol) conjugates. *Soft Matter* **2018,** *14* (30), 6320-6326.
